# A Charge‐Adhesive Targeted DNA Gel Bandage for the Precision Treatment of Inflammatory Bowel Disease

**DOI:** 10.1002/advs.202509419

**Published:** 2025-07-20

**Authors:** Peifen Lu, Hongxiu Yuan, Gang Wang, Tao Cheng, Lie Li, Yixi Dong, Runyu Zhao, Xuerui Zhang, Jianwei Jiao, Jin Jiao

**Affiliations:** ^1^ Shandong Cancer Hospital and Institute, School of Life Sciences Shandong First Medical University & Shandong Academy of Medical Sciences Jinan 250117 China; ^2^ State Key Laboratory of Stem Cell and Reproductive Biology Institute of Zoology Chinese Academy of Sciences Beijing 100101 China

**Keywords:** DNAgb, immune response regulation, inflammatory bowel disease, nuclear acid hydrogel

## Abstract

The exacerbation and recurrence of inflammatory bowel disease (IBD) are closely related to the overactivation of the immune system and the destruction of the intestinal mucosa. Current small‐molecule and biopharmaceutical therapies for IBD are often limited by off‐target effects, low bioavailability, and poor treatment outcomes, leading to systemic side effects and severe complications. To address these challenges, DNA gel bandage (DNAgb) designed to block immune cell homing and inhibit inflammatory responses are proposed. DNAgb is a negatively charged “sticky excipient” formed by a rolling circle amplification production hydrogel and an integrin α4 aptamer (ApITGA4) ‐guided tetrahedral DNA nanostructure. This unique design enables dual‐specific localization to inflamed mucosa through electrostatic interactions and ApITGA4‐mediated affinity targeting. Our studies have demonstrated that DNAgb exhibits precise targeting, superior stability, and robust anti‐inflammatory efficacy. It effectively inhibits activation of the NF‐κB signaling pathway, decreases the secretion of inflammatory factors and reshapes the immune microenvironment. Transcriptome analysis further reveals the underlying mechanism of DNAgb in IBD therapy, highlighting its role in inflammation repression. Therefore, DNAgb provides a promising strategy for local therapeutic agents that effectively inhibit the inflammatory response and provides a new and effective choice for the treatment of IBD.

## Graphical abstract

DNA gel bandage (DNAgb) is designed to precisely target the site of inflammation of IBD, by achieving dual specific localization of inflamed mucosa through electrostatic interactions and ApITGA4‐mediated affinity targeting. DNAgb effectively the activation of inflammatory pathways and reshapes the immune microenvironment. Therefore, DNAgb provides a promising strategy for local therapeutic agents that effectively inhibit the inflammatory response of IBD.

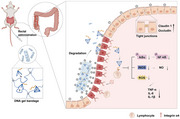

## 1 Introduction

Inflammatory bowel disease (IBD) is a common chronic gastrointestinal inflammatory disease, characterized by recurrent inflammation of the gastrointestinal tract and an increased risk of colorectal cancer.^[^ ^1^, ^2^ ^]^ The pathological mechanism of the IBD is complex and has not been fully revealed.^[^ ^3^, ^4^ ^]^ It is generally believed that IBD is related to oxidative stress, inflammation, intestinal barrier damage and intestinal microorganisms.^[^ ^5^, ^6^ ^]^ Standard clinical treatments, such as aminosalicylates, corticosteroids, and immunosuppressive agents, provide only temporary symptom relief without addressing the underlying pathogenic factors,^[^ ^7^, ^8^ ^]^ and long‐term use may cause serious complications and side effects.^[^ ^9^ ^]^ Currently IBD drugs mostly lack targeting ability and rely on blood delivery, resulting in systemic infiltration, hepatic accumulation, and low bioavailability.^[^ ^10^ ^]^ Therefore, alternative therapeutic strategies are urgently needed to accurately target the inflamed colon, restore the integrity of the intestinal barrier, reduce inflammation, and minimize systemic adverse effects. Colonic mucosal ulcer tissue is accompanied by two key properties: positively charged on the surface of the tissue^[^ ^11^ ^]^ and the specific migration of immune cells.^[^ ^12^ ^]^ The enrichment of positive charge is mainly due to the accumulation of positively charged proteins, such as: bactericidal / permeability‐increasing proteins, antimicrobial peptides.^[^ ^13^ ^]^ In addition, cationic exudates also lead to positive charges in inflammatory lesions.^[^ ^14^ ^]^ On the other hand, the tissue‐specific migration of immune cells is a key pathway that regulates IBD.^[^ ^15^ ^]^ Specifically, with the development of IBD, immune cells, including macrophages and lymphocytes, migrate to and infiltrate the site of inflammation through integrins that bind to cell adhesion molecules on endothelial cells.^[^ ^16^, ^17^ ^]^ Studies suggest that blockade of immune cell homing contributes to the excessive immune response in IBD patients and reduces inflammation and tissue damage.^[^ ^18^, ^19^ ^]^ Therefore, these features of IBD suggest that charge adsorption and integrin targeting can be used to achieve more precise drug delivery.

Tetrahedral DNA nanostructures (TDNs) represent novel nucleic acid nanomaterials with a tetrahedral spatial structure that endows them with excellent biological properties, such as enhanced cellular uptake efficiency, superior tissue permeability, and good biosafety profiles.^[^ ^20^, ^21^, ^22^ ^]^ Interestingly, previous studies have shown that TDNs possess a strong capacity in anti‐inflammation, as well as promote cell proliferation, anti‐apoptotic effects and reactive oxygen species (ROS)‐scavenging capabilities in treating a wide variety of diseases.^[^ ^23^, ^24^ ^]^ Besides, owing to their programmability and electronegativity, TDNs are potential nanostructures for IBD treatment. However, current research has focused predominantly on the intravenous injection of TDNs,^[^ ^25^ ^]^ which are susceptible to degradation due to the ubiquitous presence of nucleases in the body, thus eventually leading to a reduction in drug concentration at the lesion site, consequently compromising treatment efficacy.^[^ ^26^ ^]^ DNA hydrogels have been successfully used as artificial extracellular matrix, controlled drug delivery, capture and programmed release of bone marrow mesenchymal stem cells, and cell‐free production of protein scaffolds.^[^ ^27^, ^28^, ^29^ ^]^ For example, a rolling circle amplification (RCA)‐based DNA hydrogel is a well‐developed DNA hydrogel and is believed to have the potential to be widely used as an adjuvant therapy for many diseases.^[^ ^30^ ^]^ RCA is a simple and efficient isothermal enzyme amplification strategy for the synthesis of ultralong single‐stranded DNA.^[^ ^30^ ^]^ The compact structure of RCA‐based DNA hydrogels can reduce the degradation of DNases in intestinal fluid, thereby protecting their internal structure and enhancing their potential in IBD therapy. In addition, their long DNA strand can augment the loading capacity; moreover, the synthesis of long chains is low cost, providing advantages for the clinical application of IBD treatment.^[^ ^30^ ^]^ Therefore, the natural biocompatibility and programmability of RCA‐based DNA hydrogels are promising for their wide application in the pharmaceutical field in the future.

**Figure 1:**
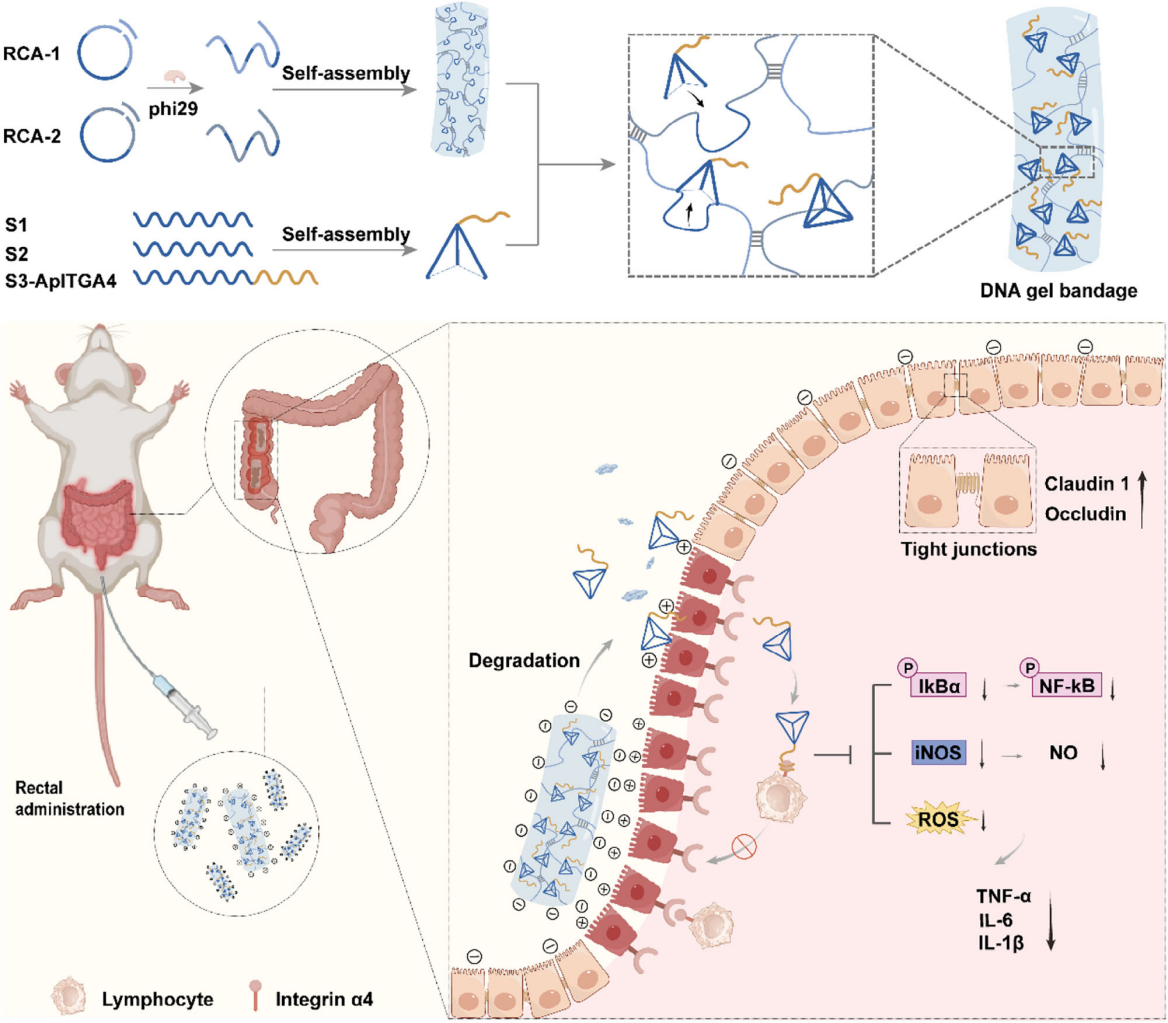
Schematic illustration of the fabrication of a DNAgb and anti‐inflammatory therapy for DSS‐induced IBD.

In this study, we report an innovative dual‐targeted DNA gel bandage (DNAgb) to alleviate IBD (**Figure** **1**). First, two ultralong DNA strands are synthesized via RCA and entangled with each other as “sticky excipients” for band aids. Although traditional TDN is assembled from four chains, the repeated fragments of RCA here serve as the fourth chain of the “trihedral DNA nanostructure”, promoting the formation and assembly of multivalent TDN units. Moreover, to further enhance targeted delivery and anti‐inflammatory capabilities, the vertex of TDN is loaded with integrin α4 aptamers (ApITGA4). The final gel state of DNAgb not only protects the functional element TDN and the aptamer but also makes the surface of DNAgb covered with negative charges because it is a component of pure DNA. After rectal administration, the DNAgb wound dressing preferentially adsorbed to the positively charged lesion site. In the complex environment of the intestine, free TDN‐ApITGA4 is subsequently gradually released. It can first block integrin α4 on immune cells from binding to the adhesion factor VCAM‐1 through ApITGA4, thereby inhibiting the inflammatory infiltration of immune cells. TDN is subsequently endocytosed into inflammatory cells to exert an inflammatory effect by blocking the NF‐κB inflammatory signaling pathway, inhibiting the secretion of inflammatory factors, eliminating ROS and NO, and inhibiting the inflammatory process. Therefore, the integrin‐targeting strategy and electrostatic interactions endow DNAgb with dual‐targeting characteristics, enhancing its accumulation at inflammatory lesion sites and protecting it from further stimulation by inflammatory cells, performing the role of a “medicinal dressing” and thereby augmenting therapeutic precision and reducing immune activation.

## 2 Results and Discussion

### 2.1 Preparation and Characterization of the DNA Gel Bandage

**Figure 2:**
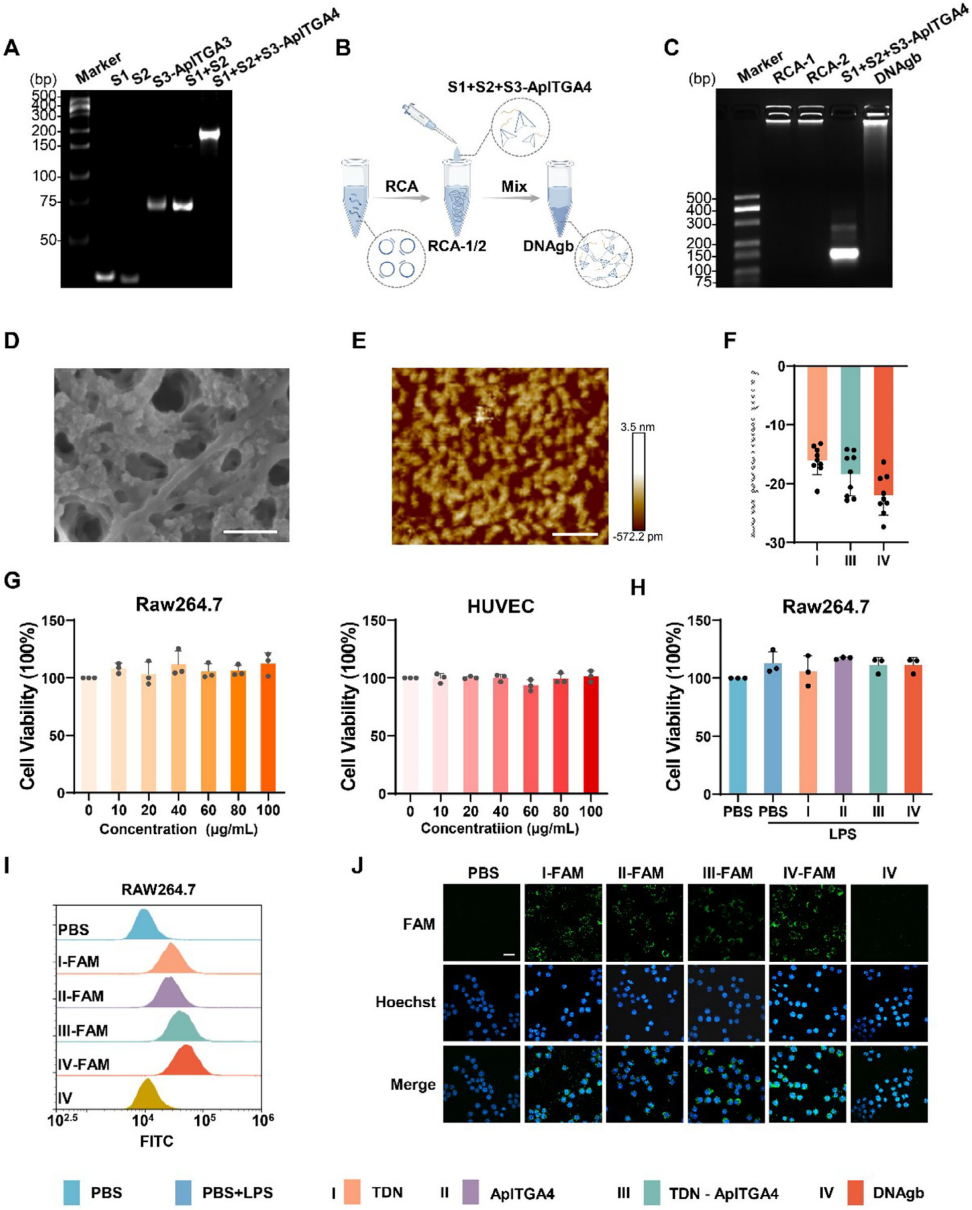
Characterization of the DNAgb. A) 10% PAGE analysis of TDN‐ApITGA4 (S1+S2+S3‐ApITGA4). B) Schematic diagram depicting the self‐assembly of DNAgb. C) 2% agarose gel electrophoresis analysis of DNAgb. D) SEM image of DNA gb; scale bar: 200 µm. E) AFM image of DNAgb; scale bar: 200 nm. F) Zeta potentials of TDN, TDN‐ApITGA4, and DNAgb. G) Biocompatibility of different nanostructures toward Raw264.7 cells and HUVECs. H) Cytotoxicity profiles of TDN, ApITGA4, TDN‐ApITGA4, and DNAgb. I) Cellular uptake efficiency of different nanostructures by Raw264.7 cells. J) Fluorescence images of the cellular uptake of different nanostructures by Raw264.7 cells; scale bar: 20 µm. The data are presented as the means ± SD, n = 3.

The DNAgb was composed of two ultralong DNA chains (including the repeated TDN S4 sequence) and an ApITGA4‐functionalized trihedral DNA nanostructure (assembled from the S1, S2, and S3‐ApITGA4 sequences). First, the trihedral DNA nanostructure was prepared by annealing with S1, S2, and S3‐ApITGA4 (**Figure** **2**A). As shown in Figure 2B, two ssDNAs were synthesized via RCA, which can hybridize and complement each other according to the circular template (circDNA‐1 and circDNA‐2) and intertwine to form a network structure (RCA‐1 + RCA‐2). Afterwards, it was hybridized with a trihedral DNA nanostructure to form a complete DNAgb Subsequently, agarose gel electrophoresis revealed that the trihedral DNA nanostructure (S1+S2+S3‐ApITGA4) was successfully synthesized and effectively combined into the gel network (Figure 2C). After that, the nanostructures of DNAgb observed by scanning electron microscopy (SEM) and atomic force microscopy (AFM) revealed an obvious network structure, indicating the successful preparation of the DNAgb (Figure 2D,E). Meanwhile, TDN (assembled from S1, S2, S3, and S4) and TDN‐ApITGA4 (assembled from S1, S2, ApITGA4‐S3, and S4) were synthesized via the same synthesis method as the trihedral DNA nanostructure. Besides, zeta potential analysis showed that the potentials of TDNs, TDN‐ApITGA4, and DNAgb were all negative, and DNAgb had the lowest potential, at ‐23.1 mV (Figure 2F). In addition, the biocompatibility and cellular uptake of DNAgb were evaluated. As shown in Figure 2G; Figure S1A (Supporting Information), the results revealed that treatment of diverse cell lines (the mouse macrophage cell line Raw 264.7, which represents inflammatory immune cells; the human umbilical vein endothelial cell line HUVEC, which represents normal human endothelial cells; and the human colorectal cancer cell line Caco2, which is a commonly used epithelial tissue model of the colon) with DNAgb caused very little cytotoxicity after 24 h of culture. Similarly, DNAgb did not affect Raw264.7 cells in the LPS‐stimulated inflammatory state (Figure S1B, Supporting Information). In addition, TDN, ApITGA4, TDN‐ApITGA4, and DNAgb were applied to Raw264.7 cells with or without LPS stimulation, and cell viability was determined. As shown in Figure 2H and Figure S1C (Supporting Information), the results demonstrated that cell viability was not affected, indicating that these DNA structures exhibited excellent biocompatibility. To evaluate the cellular uptake behavior, FAM‐labeled TDN, ApITGA4, TDN‐ApITGA4, and DNAgb were prepared to understand the internalization by macrophages via flow cytometry analysis. Results revealed that TDN, TDN‐ApITGA4 and DNAgb can be effectively taken up by cells (Figure 2I). Similarly, the results of confocal laser scanning microscopy also proved the same results. It was worth mentioning that compared with TDNs, ApITGA4, TDN‐ApITGA4, and DNAgb presented stronger fluorescence signals, which may be due to their large structures that were more easily internalized by cells.

### 2.2 In Vitro Validation of the Anti‐Inflammatory Effect of DNA Gel Bandage

**Figure 3:**
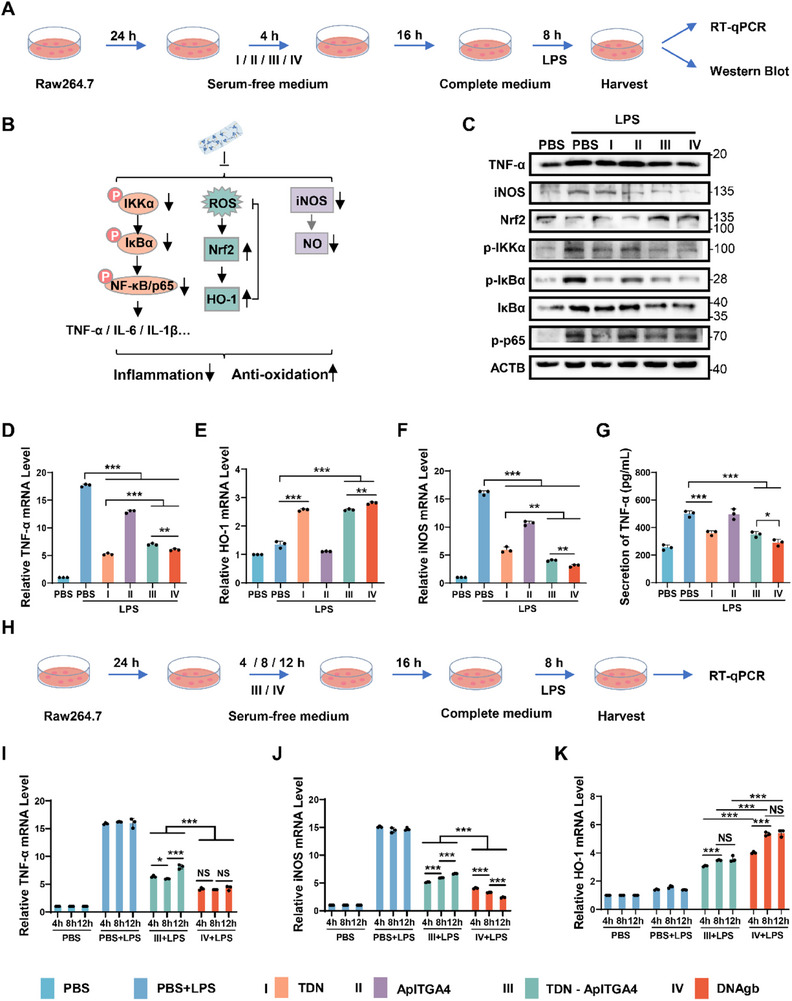
In vitro anti‐inflammatory effect of a DNAgb. A) Illustration of the cell experiment. B) Schematic diagram of key inflammatory signal regulators involved in the anti‐inflammatory effect of DNAgb. C) Western blotting analysis of analyzed the protein expression of related inflammatory factors in Raw264.7 cells after different treatments. The mRNA expression of TNF‐α D), iNOS E), and HO‐1 F) in Raw264.7 cells. G) TNF‐α expression in Raw264.7 cell supernatants after different treatments. The mRNA expression of TNF‐α H), iNOS I), and HO‐1 J) in Raw264.7 cells after different treatments under different increasing treatment time. The data are presented as the mean ± SD, n = 3. Statistical analysis was performed via Student's t test. *P < 0.05, **P < 0.01, ***P < 0.001.

To evaluate the therapeutic potential of DNAgb, we first evaluated its anti‐inflammatory effect at the cellular level. Raw264.7 cells were stimulated with LPS to mimic the conditions of cells in the inflamed intestinal environment. As shown in **Figure** **3**A, B, after treatment with TDN, ApITGA4, TDN‐ApITGA4, or DNAgb, the cells were stimulated with LPS for 8 h, and then the cells and media were collected to examine the levels of inflammatory factors and key regulators. Previous studies reported that TDN can inhibit the inflammatory process by repressing the NF‐κB signaling pathway, decreasing the secretion of inflammatory factors, and scavenging ROS and NO.^[^ ^31^, ^32^ ^]^ Among the treatments, DNAgb had the most pronounced anti‐inflammatory effects, significantly reducing TNF‐α levels and inhibiting key regulators of NF‐κB signaling, including p‐IKKα, p‐IκBα, IκBα and p‐p65 (Figure 3C). DNAgb also downregulated inducible nitric oxide synthase (iNOS) expression while upregulating Nrf2, indicating the activation of ROS scavenging pathways (Figure 3C). Moreover, the qRT‒PCR results revealed that, compared with the control, TDN‐ApITGA4 significantly decreased the mRNA expression of the inflammatory factors TNF‐α, IL‐6, and IL‐1β in Raw264.7 with LPS challenge, as compared to the controls (Figure 3D; Figure S2, Supporting Information). In contrast, the mRNA level of iNOS was significantly decreased under TDN‐ApITGA4 and DNAgb treatment, and the level of heme oxygenase‐1 (HO‐1) (a downstream target of Nrf2) was apparently increased under TDN‐ApITGA4 and DNAgb treatment (Figure 3E, F). Notably, compared with TDN‐ApITGA4, DNAgb exhibited better anti‐inflammatory ability through further inhibition of the expression of those inflammatory factors and key regulators. In addition, the secretion of TNF‐α was further determined by ELISA, and the results showed that it was highly inhibited, which was consistent with the results of western blotting and qPCR (Figure 3G). Taken together, these findings confirmed that DNAgb has excellent anti‐inflammatory effects.

Furthermore, considering the different stabilities of TDN‐ApITGA4 and DNAgb, we speculated that DNAgb has advantages in the anti‐inflammatory effects in long‐term therapy. Therefore, we extended their coincubation time with cells and subsequently examined the fluorescence stability and the mRNA levels of the relevant key regulators (Figure S3A, Supporting Information). Flow cytometry experiments demonstrated that cells treated with DNAgb presented greater fluorescence intensity than did those treated with TDN‐ApITGA4 over time (Figure S3B, Supporting Information). In addition, DNAgb‐treated cells presented more durable and stable inflammation inhibition (Figure 3H–J).

Collectively, these findings suggest that DNAgb has an efficient anti‐inflammatory effect on macrophages, suggesting its potential therapeutic efficacy in IBD treatment.

### 2.3 Spatiotemporal Distribution of DNA Gel Bandage In Vivo

**Figure 4:**
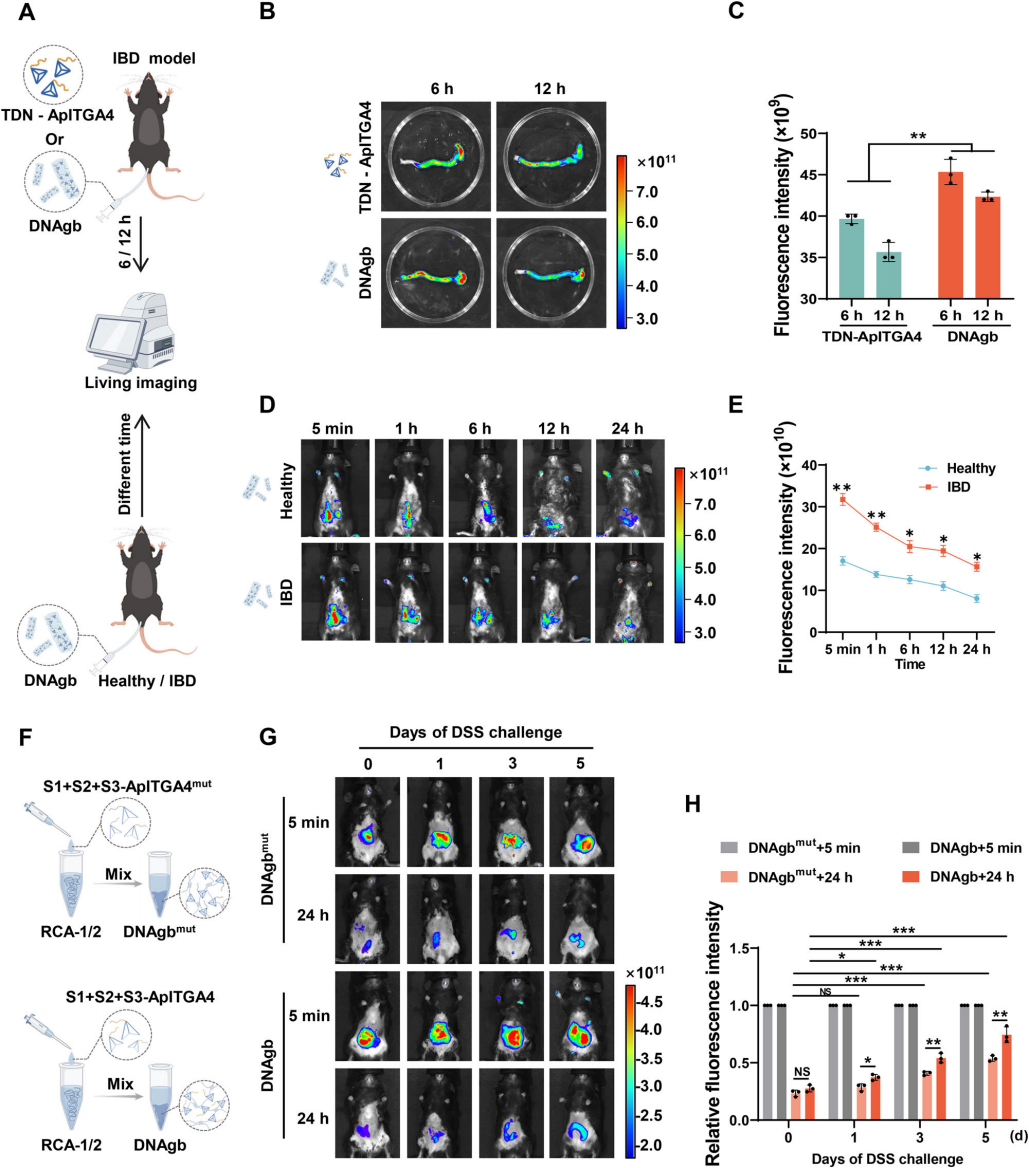
Spatiotemporal distributions of DNAgb. A) Schematic illustration of the establishment and treatment schedule of healthy/IBD mice. B, C) After IBD mice were treated with TDN‐ApITGA4 (Cy5‐labeled) and DNA gb (Cy5‐labeled) for 6 h and 12 h, the colons were imaged with an in vivo imaging system, and the Cy5 fluorescence signal was quantified. D, E) Fluorescence images of healthy and IBD mice at different time after rectal administration of DNAgb and quantification of the Cy5 fluorescence signal. F) Schematic illustration of DNAgb^mut^ and DNAgb synthesis. G, H) Fluorescence images of healthy and different DSS‐challenged mice at 5 min and 24 h after rectal administration of DNAgb^mut^ or DNAgb and quantification of the Cy5 fluorescence signal. DNAgb^mut^, DNAgb with the ApITGA4 mutation. The data are presented as the mean ± SD, n = 3. Statistical analysis was performed using student's t‐test. *P < 0.05, **P < 0.01, ***P < 0.001.

Considering that effectively targeting DNAgb is the key to ensuring its therapeutic effect on IBD, its spatiotemporal distribution was measured in vivo. DNAgb is designed as a dual‐targeted structure with an aptamer and charge attraction to prolong its residence time in inflammatory lesions. To test this hypothesis, we constructed a mouse model of IBD to study the dual targeting effect in vivo. IBD mice were induced by continuously administering 3.5% (w/v) dextran sulfate sodium (DSS) in the drinking water for five days and then treated with TDN‐ApITGA4 and DNAgb via rectal administration (**Figure** **4**A). After 6 h or 12 h, the mice were sacrificed, and the colons were isolated for fluorescence detection. As shown in Figure 4B, C, the fluorescence intensity observed in the image of the DNAgb was significantly greater than that of TDN‐ApITGA4 in the colon at 6 h and 12 h after administration. In addition, the stability of TDN‐ApITGA4 and DNAgb in simulated intestinal fluid (SIF) was measured. The data revealed that in SIF, TDN‐ApITGA4 started to degrade on the second day, whereas the DNAgb remained stable on the third day, indicating that the DNAgb maintained effective adhesion in the colon and protected it (Figure S4, Supporting Information). To evaluate the ability of DNAgb to target inflammatory sites, healthy mice and DSS‐treated mice were subjected to rectal administration of DNAgb, and the temporal dynamic fluorescence intensity was measured (Figure 4A). The fluorescence intensity of the DSS‐treated mice was significantly greater than that of the healthy mice, and fluorescence was still observed in the DSS‐treated mice after 24 h of treatment (Figure 4D,E).

To investigate whether there is a synergistic effect between ApITGA4 and charge attraction in targeting colitis sites, we synthesized ApITGA4‐mutant DNA hydrogels (DNAgb^mut^) to eliminate the integrin‐targeting effect (Figure 4F). Flow cytometry revealed that the mutated ApITGA4 (ApITGA4^mut^) could not bind to Raw264.7 cells (Figure S5, Supporting Information). We established different degrees of inflammation in the IBD model on different DSS challenge days (0, 1, 3, and 5 days), and the results revealed different positive charge intensities in the colon. Then, DNAgb^mut^ and DNAgb was rectal administrated and fluorescence imaging was performed. As shown in Figure 4G, H, the relative fluorescence intensity of DNAgb was significantly greater than that of DNAgb^mut^ in mice subjected to different durations of DSS challenge, and the difference increased with increasing DSS challenge duration, indicating that ApITGA4 could increase the enrichment of inflammation sites, with greater enrichment alongside inflammation severity. In addition, the relative fluorescence intensities of the DNAgb^mut^ and DNAgb treatments increased with the number of days of DSS induction, suggesting the role of electrostatic adsorption in inflammatory targeting. To further confirm the synergistic effect between ApITGA4 and charge attraction, we then performed charge neutralization experiments with DNAgb^mut^ and DNAgb. MgCl_2_ was reported to be suitable for neutralizing the charge of DNA materials.^[^ ^33^ ^]^ Under 125 mM MgCl_2_ environment, the zeta potential of DNAgb^mut^ and DNAgb. showed an almost neutral charge (Figure S6, Supporting Information). We further explored their targeting efficiency in IBD mice. The results revealed that the relative fluorescence intensity of the mice treated by DNAgb^mut^ or DNAgb supplemented with MgCl_2_ was significantly lower than that of the mice treated with DNAgb alone (Figure S7, Supporting Information). Notably, when the mice were treated with DNAgb^mut^ supplemented with MgCl_2_, the relative fluorescence intensity was dramatically inhibited, indicating a synergistic effect between these two processes (Figure S7, Supporting Information). Taken together, these observations suggest that the dual‐targeting properties of DNAgb prolong its residence time within the colon.

### 2.4 Therapeutic Efficacy of DNA Gel Bandage in DSS‐Induced IBD Model Mice

**Figure 5:**
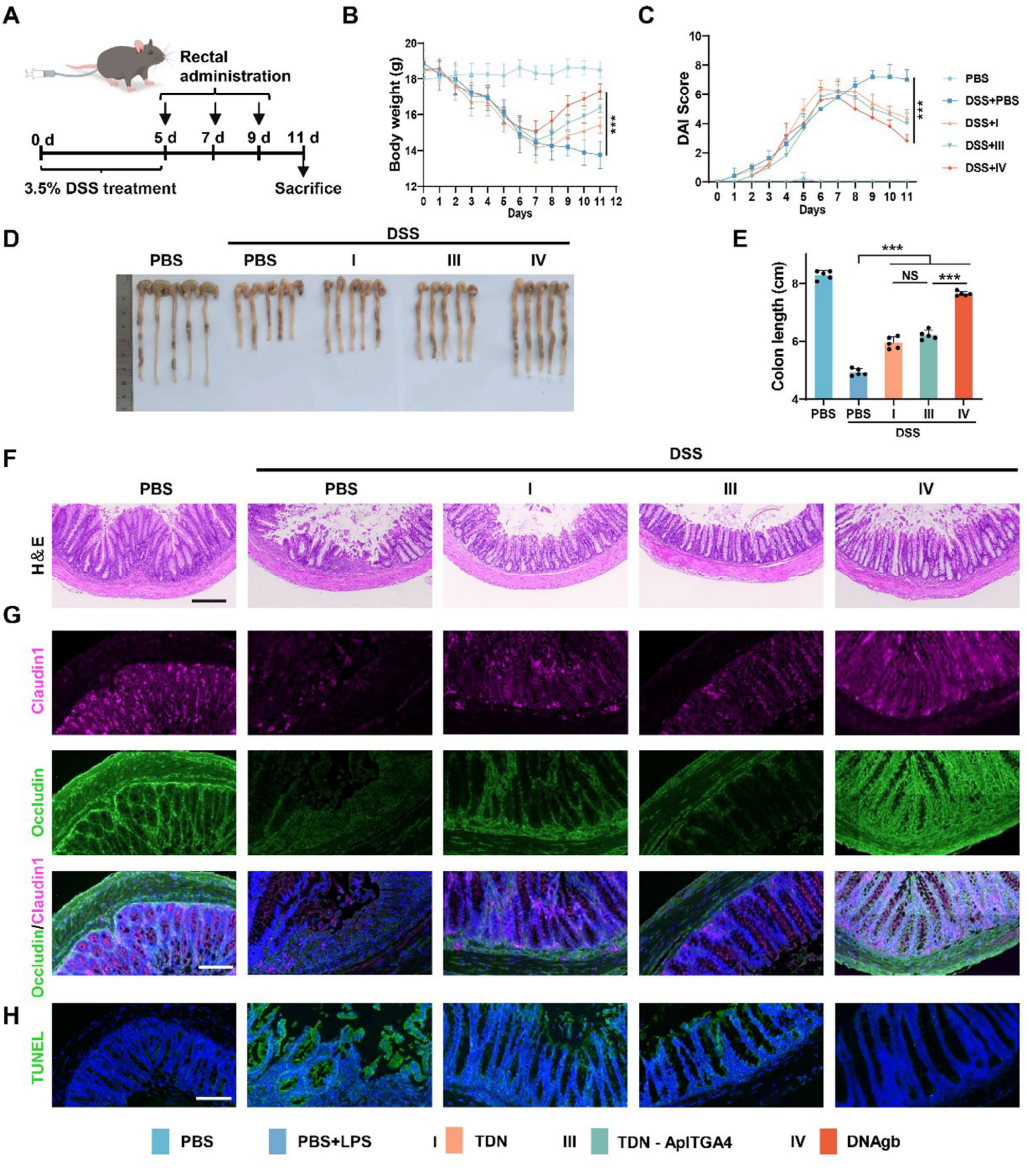
Therapeutic efficacy of a DNAgb in DSS‐induced IBD model mice. A) Experimental flowchart of DNAgb treatment of DSS‐induced IBD mice. B) Daily body weight changes in each group. C) Disease activity index (DAI) scores changed during the experiment in each group of mice. D) Digital photographs of the colons of each group of mice on day 11. E) Statistical analysis of colon length. F) H&E staining of colon tissue sections from each group of mice; scale bar: 100 µm. G) Immunofluorescence staining of colon sections was used to observe the expression of various mouse intestinal tight junction proteins, such as claudin 1 (red) and occludin (green); scale bar: 100 µm. H) TUNEL images of colon tissue from each group; scale bar: 100 µm. The data are presented as the mean ± SD, n = 5. Statistical analysis was performed via two‐way ANOVA. ***P < 0.001.

On the basis of the advantages of the anti‐inflammatory effects of DNAgb in vitro and colonic retention in vivo of DNAgb, we further evaluated its therapeutic effect on IBD. mice were exposed to 3.5% DSS in the drinking water for 5 days, followed by three times rectal administration of various DNA structures every two days (**Figure** **5**A). As shown in Figure 5B, the body weights of the IBD mice after treatment with DNAgb were similar to those of the normal control mice. The DNAgb group also presented a lower disease activity index (DAI) (Figure 5C). In addition, after treatment with DNAgb, the colon length also recovered close to that of normal mice (Figure 5D, E). These results clearly demonstrate the strong preventive effect of DNAgb on colitis.

The therapeutic efficacy of DNAgb was further evaluated via histological analysis. Hematoxylin and eosin (H&E) staining revealed that the intestinal mucosa of IBD model mice was damaged, manifested as rupture, separation, inflammatory cell infiltration, and severe damage to the colonic crypt. While the DNAgb treatment group was relatively complete (Figure 5F). In addition, we assessed the intestinal barrier integrity of two tight junction proteins, Claudin1 and Occludin, which are responsible for enclosing the paracellular space between intestinal epithelial cells. The immunohistochemistry (IHC) results revealed much greater expression of Occludin and Claudin1 in the IBD mice treated with DNAgb (Figure 5G). In addition, terminal deoxynucleotidyl transferase‐mediated deoxyuridine triphosphate nick end labeling (TUNEL) staining revealed a reduced fluorescence signal in IBD mice treated with DNAgb, indicating that apoptosis at the inflammatory site was significantly reduced (Figure 5H). Moreover, the ROS level was confirmed by dihydroethidium staining, and the results confirmed that the ROS level of DNAgb was sharply downregulated in the DNAgb group, further revealing that DNAgb can promote ROS clearance (Figure S8, Supporting Information). Finally, after treatment with different DNA structures, there was no significant change in the tissue morphology of the main organs, indicating that it had good biosafety (Figure S9, Supporting Information). These findings demonstrated the potential of DNAgb as a promising therapeutic intervention for alleviating acute DSS‐induced IBD.

### 2.5 Transcriptome Analysis after DNA Gel Bandage Treatment

**Figure 6:**
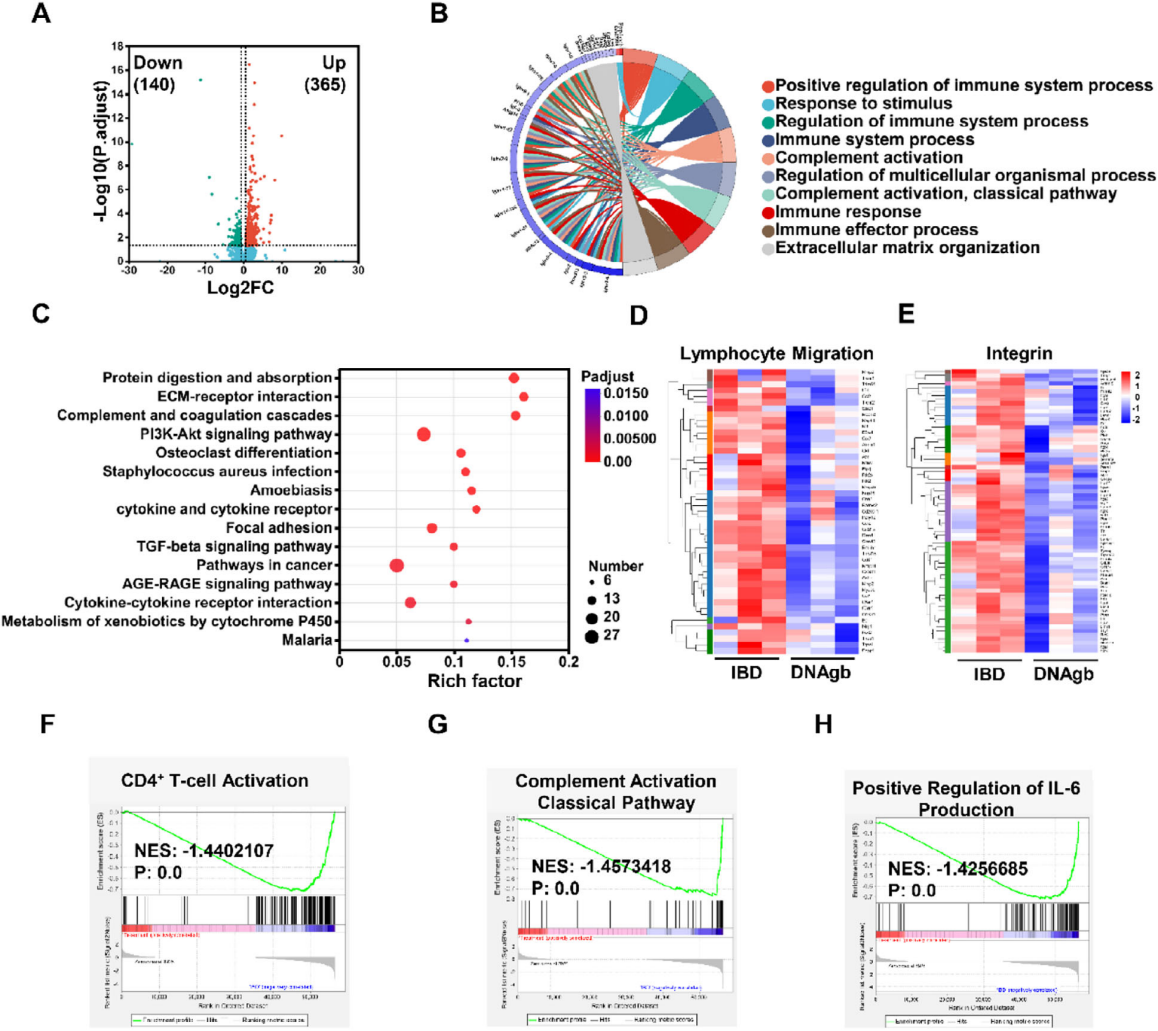
Transcriptomic analysis of DSS‐induced colitis regulated by DNAgb. A) Volcano plot of the differentially expressed genes in the colon tissues of the control DSS and DNAgb. B) GO biological process clustering analysis. C) KEGG pathway enrichment analysis of DEGs in the colitis group versus the DNAgb‐treated group. D) Heatmap of the genes involved in lymphocyte migration (D) and Integrin related pathway E) in the DSS and DNAgb groups. GSEA of gene sets related to “CD4^+^ T‐cell activation” F), “complement activation classical pathway” G), and “positive regulation of IL‐6 production” H). NES, normalized enrichment score. |NES| > 1 and p value < 0.05 were considered statistically significant in the PBS group versus the DNAgb group.

To investigate the therapeutic mechanism involved in the treatment of IBD via DNAgb, RNA sequencing was performed to comprehensively and quantitatively evaluate the gene expression landscape in colon tissue. Differential expression analysis revealed 365 upregulated and 140 downregulated genes in the DNAgb‐treated IBD mice compared with the control mice (**Figure** **6**A). Subsequently, the differential expression genes (DEGs) were subsequently clustered for enrichment analysis to reveal the therapeutic mechanism of DNAgb. First, the results of the gene ontology (GO) analysis revealed that the DEGs were related to several biological pathways, including active regulation of immune system processes, complement activation classical pathways, and immune effectors, etc. (Figure 6B). Moreover, Kyoto Encyclopedia of Genes and Genomes (KEGG) analysis revealed that DNAgb treatment mainly affected signaling pathways related to extracellular matrix (ECM) – receptor interaction, complement and coagulation cascades, the PI3K‒Akt signaling pathway, the cytokine‒cytokine receptor interaction pathway, etc. (Figure 6C). Among them, immune system‐related processes are consistent with the performance of DNAgb in vitro and in vivo, and ECM biology has been reported to involve complex interconnections with immune cells.^[^ ^34^ ^]^ The PI3K‐Akt signaling pathway is an important signaling pathway for regulating apoptosis, suggesting that it may be the mechanism of decreased intestinal epithelial cell apoptosis after DNAgb treatment. To further characterize the therapeutic effect of the DNAgb, we analyzed the expression profiles of specific pathway gene sets. As shown in Figure 6D, E, the heatmap revealed that, compared with those in the DSS treatment group, the expression of genes related to lymphocyte migration and the integrin pathway was significantly downregulated after DNAgb treatment, highlighting the role of lymphocyte blockade of the ApITGA4 element within DNAgb. To reduce the analysis bias caused by threshold selection, we further performed gene set enrichment analysis (GSEA). As shown in Figure 6F–H, DNAgb treatment was also related to several pathways, including those related to the activation of CD4^+^ T‐cell activation, complement activation, and IL‐6 production, highlighting the potential role of DNAgb in inflammatory immune remodeling.

### 2.6 Regulatory Effects of DNA Gel Bandages on Immune Responses in DSS‐Induced IBD Model Mice

**Figure 7:**
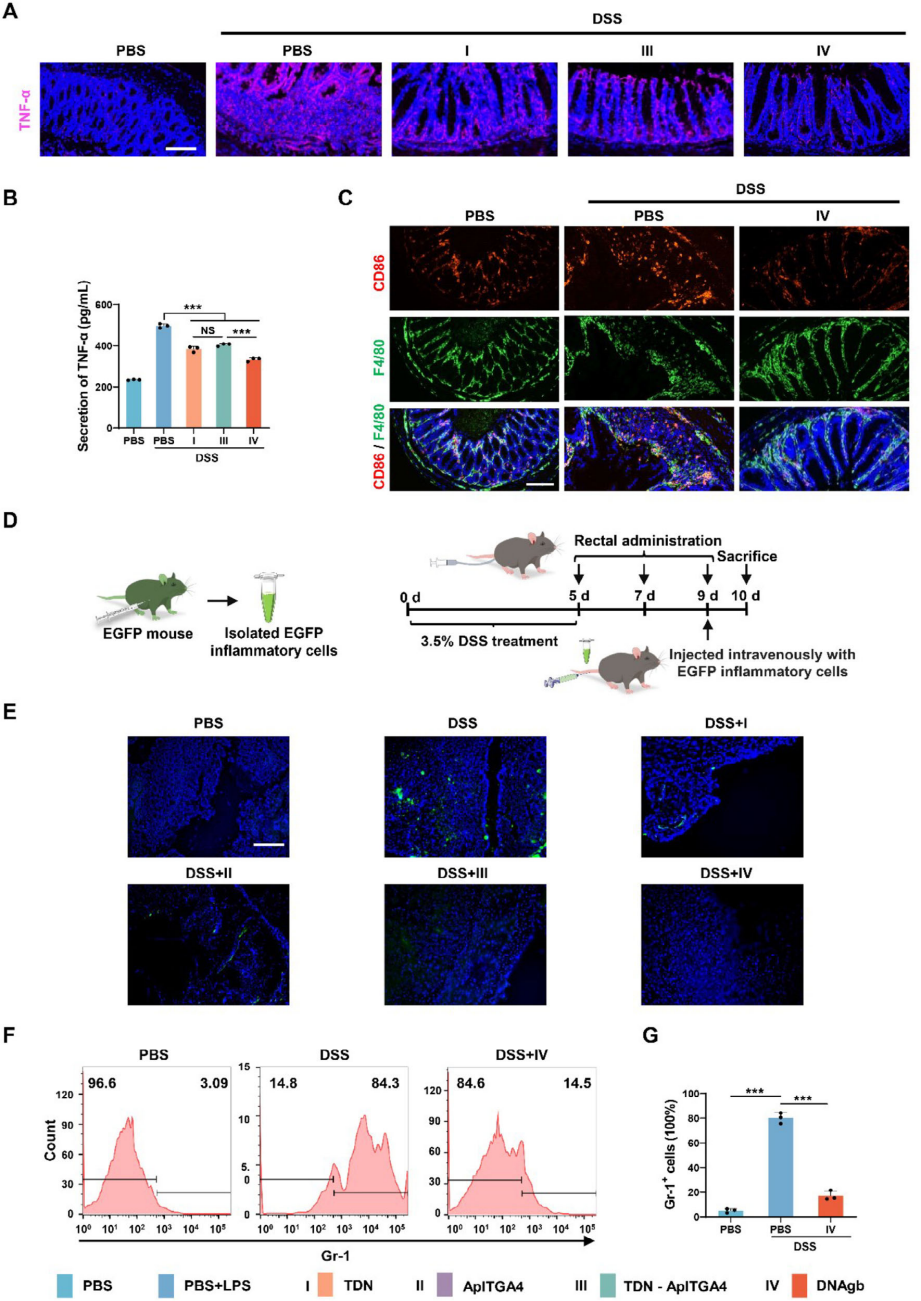
Regulatory effects of DNAgb on immune responses in DSS‐induced IBD model mice. A) Immunofluorescence staining of two key molecules of the inflammatory pathway, TNF‐α, under different treatments. Scale bar: 100 µm. B) ELISA results showing the level of TNF‐α in colon tissues from IBD mice subjected to different treatments. C) Immunofluorescence staining of M1 macrophage subtypes (F4/80^+^ CD86^+^) in colon tissues from IBD mice subjected to different treatments. D) Experimental flowchart of the isolation of EGFP‐expressing inflammatory cells from the abdominal fluid of EGFP‐expressing mice (left panel) and IBD mice subjected to different pretreatments and injected intravenously with EGFP‐expressing inflammatory cells. E) Colon sections showing EGFP‐induced inflammatory cell infiltration. Scale bar: 50 µm. F, G) Flow cytometric analysis of neutrophils from the colonic mucosa of IBD mice subjected to DNAgb treatment, and statistics showed the proportion of Gr‐1 cells in total lympho‐monocytes collected from colonic tissues. The data are presented as mean ± SD, n = 3. ***P < 0.001.

After confirming its therapeutic effects and completing a preliminary mechanistic study via RNA‐seq, we investigated the regulation of immune responses by DNAgb in IBD mice. First, we evaluated the TNF‐α level in colon tissues. As shown in **Figure** **7**A, IHC revealed that DNAgb significantly inhibited TNF‐α levels in colon sections. In addition, the mRNA and protein levels of TNF‐α also revealed significant inhibition by DNAgb in the colon tissue of IBD mice (Figure 7B; Figure S10A, Supporting Information). Moreover, the mRNA levels of other cytokines, including IL‐6 and IL‐1β, consistently exhibited the same expression trend (Figure S10B, C, Supporting Information). To further investigate the effect of DNAgb on macrophages, we next measured the role of DNAgb in reshaping immune homeostasis by driving macrophage polarization. The colonic epithelium of colitis mice is usually enriched with a large number of CD86 macrophages (type M1) and few CD206 macrophages (type M2), representing the occurrence of inflammation. Here, through immunofluorescence staining, macrophages were labeled with F4/80, and M1 / M2 macrophages were identified by CD86 / CD206, respectively. Intuitively, as shown in Figure 7C and Figure S11 (Supporting Information), DNAgb was found to remarkably increase the ratio of M2 – type macrophages (F4/80^+^ CD206^+^), and to decrease the ratio of M1‐ type macrophages (F4/80^+^ CD86^+^ cells), indicating DNAgb treatment facilitated the macrophage phenotype polarization from M1 to M2. Taken together, these results indicate that DNAgb treatment exerts anti‐inflammatory effects via regulating the polarization of macrophages in IBD mice.

Given the important role of inflammatory cell homing in IBD development and considering that integrins act as “grippers” for inflammatory cell homing, we investigated the effect of DNAgb, which carries the aptamer of integrin ApITGA4, on inflammatory cell homing in IBD mice. As shown in Figure 7D, we first prepared EGFP‐producing inflammatory cells in the abdominal fluid of EGFP‐expressing mice. Moreover, healthy and IBD mice were pretreated with different DNA materials and injected intravenously with EGFP‐expressing inflammatory cells. Finally, the mice were sacrificed, and colon sections were used to analyze the infiltration of EGFP‐expressing inflammatory cells. The data revealed that the infiltration of EGFP‐expressing inflammatory cells into the colons of the mice treated with ApITGA4, TDN‐ApITGA4, or DNAgb was effectively inhibited (Figure 7E). Interestingly, mice treated with TDN, although as no ApITGA4 linked, also repressed the infiltration of inflammatory cells, which may be caused by the relief of the inflammation (Figure 7E). Notably, the TDN‐ApITGA4 and DNAgb treatment groups presented particularly significant inhibition of EGFP inflammatory cell infiltration, which was almost equivalent to that of healthy controls, suggesting a synergistic effect of ApITGA4 with TDN (Figure 7E).

Furthermore, to explore the remodeling of the immune microenvironment by DNAgb, we measured the content of T cells and neutrophils in the colonic mucosa of IBD mice after DNAgb treatment. Flow cytometric analysis revealed that the colonic mucosa of IBD mice had lower TCRαβ^+^ T cells infiltrated after DNAgb treatment, as well as Gr‐1^+^ (neutrophil marker) cells, indicating that DNAgb could reshape the immune microenvironment by repressing the infiltration of T cells and neutrophils in the colonic mucosa of IBD mice (Figure 7F, G; Figure S12, Supporting Information).

Taken together, these results revealed the role of DNAgb in immune responses in DSS‐induced IBD model mice through the inhibition of the homing of inflammatory cells and immune cell infiltration.

## 3 Conclusion

In this study, a dual‐target DNAgb was developed for precise targeting and efficient therapeutic effects on IBD by blocking inflammatory signaling pathways and inhibiting the secretion of inflammatory factors. It can be anchored to inflamed mucous membranes, with electrostatic attraction and affinity targeting of ApITGA4, to further increase bioavailability. Our findings demonstrate that DNAgb not only suppresses the secretion of inflammatory cytokines (TNF‐α, IL‐6, and IL‐1β) and scavenges ROS and NO but also helps restore the intestinal barrier. Moreover, it also plays a role in positively regulating immune system processes through immune cells blocking the ApITGA4 element. In summary, DNAgb represents a promising strategy for local therapeutic interventions, offers targeted, efficient, and safe inflammation control, and highlights a new view of the application of pure DNA nanostructures in disease treatment.

## 4 Experimental Section

### Materials

Phi 29 DNA polymerase (10 U/µL) and T4 DNA ligase were purchased from Thermo Fisher Scientific. DMEM was purchased from Gibco. Raw264.7 and Caco2 were obtained from the Cell Bank/Stem Cell Bank, Chinese Academy of Sciences (Raw264.7, Cat: SCSP‐5036, RRID: CVCL_0493; Caco2, Cat: SCSP‐5027, RRID: CVCL_0025); HUVEC was obtained from ATCC (Cat: CRL‐1730, RRID: CVCL_9Q53). All the cell lines were contamination free. Cell cryopreservation solution was obtained from Biochannel Biological Technology. Dextran sodium sulfate (MW: 36000‐50,000 Da) was purchased from MP Biomedicals Inc. TNF‐α enzyme‐linked immunosorbent assay (ELISA) kit was purchased from Shanghai Enzyme Biotechnology Co., Ltd. RNA extraction kit was obtained from Keyoubo Biotechnology Co., Ltd. Female mice were purchased from the Laboratory Animal Center of Shandong First Medical University (Jinan, China). All animal experiments were approved by the Ethics Committee of Shandong First Medical University. All oligonucleotides were synthesized by Sangon Biotechnology Co., Lid. (Shanghai, China) and GENCEFE Biotech (Wuxi, China).

### Synthesis of Circular DNA and RCA Products

The linear DNA template (10 µM) and DNA primer (10 µM) were mixed at a ratio of 1:5. Then mixture was cycled with the following procedure: denaturation at 95 °C for 5 min, cooling to room temperature gradually. The produce was incubated at 22 °C for 2 h with T4 DNA ligase and T4 DNA ligase buffer and then incubated at 65 °C for 10 min to inactivate T4 DNA ligase. RCA reaction was performed as described previously.^[^ ^35^ ^]^ Briefly, circ‐DNA 1 /2 (50 nM) and dNTPs (1 mM) was mixed with phi29 DNA polymerase (150 U/mL) and pyrophosphatase (0.002 U/µL) in a total volume of 100 µL. And then incubated in a thermoshaker at 37 °C, 450 rpm for 2 h. This mix was then heated at 75 °C for 10 min for phi29 polymerase inactivated. And RCA products were purified through centrifugation (7500 rpm, 10 min) with 30 kDa MWCO ultrafiltration device (Merck Millipore). Sequences were listed in Table S1 (Supporting Information).

### Synthesis of DNA Gel Bandage

First, trihedral DNA nanostructure was prepared by annealed with S1, S2, and S3‐ApITGA4 at a ratio of 1:1:1, with denaturation the following procedure: denaturation at 95 °C for 10 min, cooling to room temperature gradually. For the synthesis of the DNA gel bandage (DNAgb), RCA‐1 and RCA‐2 were mixed with trihedral DNA nanostructure at a ratio of 1:1:10 and incubated for 10 min at 95 °C, and cooled to 37 °C for 4 h,300 rpm. Sequences were listed in Table S1 (Supporting Information).

### Synthesis of TDN and TDN‐ApITGA4

TDN was prepared by annealed with S1, S2, S3, and S4 at a ratio of 1:1:1:1, with denaturation the following procedure: denaturation at 95 °C for 10 min, cooling to room temperature gradually. The synthesis of TDN‐ApITGA4 used same procedure but replace S3 with S3‐ApITGA4.

### PAGE

In order to demonstrate that TDN‐ApITGA4 was synthesized as expected, 10% native PAGE gel was utilized to compare and confirm the molecular mass of ssDNA and TDN‐ApITGA4 with its theoretical value.

### Agarose Gel Electrophoresis

2% Agarose gel electrophoresis was used to characterize the synthesis of DNAgb. The sample was composed of nucleic acid samples, Gel Red dye, and loading buffer. Scanning 30 min at 120 V using the Chemi Scope 6200 fluorescence chemiluminescence gel imaging system (Shanghai Qinxiang Scientific Instrument Co., Ltd).

### SEM

All the samples were put on the silicon wafer and rapidly frozen in liquid nitrogen. After drying in a vacuum, the samples were sprayed with gold and observed using a scanning electron microscope (SEM; HITACHI Regulus8100, Japan).

### Cell Culture

Raw264.7 and HUVEC were cultured in DMEM, and supplemented with 10% fetal bovine serum (FBS) (VivaCell, Shanghai) and 1% antibiotic‐antimycotic. And Caco2 cells were cultured in DMEM supplemented with 20% fetal bovine serum (FBS) and 1% antibiotic‐antimycotic. All cells were cultured at 5% CO_2_ atmosphere at 37 °C.

### Flow Cytometry

After a 24 h culture in 12‐well plates, Raw264.7 cells were treated with TDN, TDN‐ApITGA4 and DNAgb for different time. Then cells were collected washed, and resuspended in PBS. Then analyzed by flow cytometry (NovoCyte‐A310, Agilent Biosciences Co., Ltd, Hangzhou).

### Confocal Imaging

To evaluate the cellular uptake directly, a confocal microscope (Zeiss Celldiscoverer 7, Germany) was used to test. Cells were incubated with FAM‐labeled TDN, FAM‐labeled ApITGA4, FAM‐labeled TDN‐ApITGA4, FAM‐DNAgb, and no labeled DNAgb as the control for 4 h. Then cells were stained with Hoechst and fixed paraformaldehyde solution.

### MTT

Raw264.7 cells, HUVEC cells and Caco2 cells were cultured for 24 h and treated with 0, 10, 20, 40, 60, 80, 100 µg mL^−1^ DNAgb. After one day, the medium was discarded and incubated with 10 µL MTT (5 mg mL^−1^) (TargetMol, USA) for 4 h. DMSO was added and the optical density was measured at 490 nm. Furthermore, Raw264.7 was added to TDN, ApITGA4, TDN‐ApITGA4 and DNAgb of the same concentration and the absorbance intensity at 490 nm was detected by the microplate reader (TECAN, Infinite E Plex) to calculate cell viability.

Raw264.7 cells were incubated with equal concentration of TDN, ApITGA4, TDN‐ApITGA4 and DNAgb and various concentrations of DNAgb respectively after LPS stimulation. Then MTT was used to analyze cell viability.

### ELISA

The cells were incubated with TDN, ApITGA4, TDN‐ApITGA4, and DNAgb for 4 h and then induced with LPS for 8 h, followed by collecting the supernatant. The expression of TNF‐α was measured by enzyme‐linked immunosorbent assay (ELISA) kits according to the manufacturer's protocols.

### Real‐Time Quantitative PCR

Raw264.7 cells were seeded in a 6‐wells plate and cultured for 24 h; 4 h after incubating with TDN, ApITGA4, TDN‐ApITGA4, and DNAgb, the cells were induced by LPS for 8 h. Total RNA was extracted from cells using a commercially available RNA extraction kit. Complementary DNA (cDNA) was reverse‐transcribed to cDNA. The TNF‐α, HO‐1 and iNOS gene expressions were measured by ChamQ SYBR Color qPCR Master Mix (Vazyme, Nanjing, China) in Roche LightCycler 480 Real‐Time PCR System (Roche, Switzerland). ACTB was used as an internal control gene, and analyzed according to the 2^−∆∆Ct^ method. Primers were shown in Table S2 (Supporting Information).

### Western Blot Analysis

After drug treatment and LPS stimulation, the total protein of Raw264.7 cells was extracted using a Western and IP lysis kit (Beyotime, Beijing) with protease and phosphatase inhibitor cocktails (APExBIO Technology LLC, USA). Protein quantification was performed using the BCA protein assay kit (Smart‐Lifesciences). And the protein samples were desaturated at 100 °C for 10 min with SDS loading buffer (Genscript Biotech, Nanjing). Then SDS‐PAGE was used to separate the samples. Subsequently, the gel was transferred to a PVDF membrane, blocked with 5% skimmed milk and incubated with primary and secondary antibodies. Immunoreactivity was observed with gel imaging system (QinXiang, ChemiScope 6000). The primary antibody including: TNF‐α antibody (HuaBio, Hangzhou), iNOS (HuaBio, Hangzhou), Nrf2 antibody (Abcam, Britain), p‐IKKα (HuaBio, Hangzhou), p‐IκBα antibody (HuaBio, Hangzhou), IκBα antibody (HuaBio, Hangzhou), p‐p65 antibody (Bioworld, Nanjing), and ACTB antibody (ABclonal, Wuhan).

### Fluorescence Imaging and Biodistribution

Mice were rectal administrated with different Cy5‐labbled DNA materials and imaged using an in vivo imaging system at different time points. For colon biodistribution imaging, the mice were sacrificed at predetermined time points (6 h and 12 h) after rectal administration and gathered colons to scan using an in vivo imaging system (Tanon, ABL‐X5).

### DSS‐Induced Colitis Mice Modeling

C57BL/6 mice (female, 6‐weeks old) were randomly divided into the control group, the DSS+PBS group, the DSS+TDN group, the DSS+ApITGA4 group, the DSS+TDN‐ApITGA4 group and the DSS+DNAgb group. The control group was given sterile water while others were given 3.5% w/v DSS water for 5 days to establish IBD model. After, mice were rectal administration with different DNA materials at 100 µl (3 µmol L^−1^) once two days. During the period, the fecal consistency, fecal blood, and weight loss of mouse were recorded daily to determine changes in the DAI index. At the end of 11 days, all mice were euthanized, colons were harvested and analyzed. The animal studies were approved by the Ethics Review Committee of Shandong First Medical University (W202406030482), and all the protocols conformed to the guidelines for ethical conduct in the care and use of nonhuman animals in research.

### Histopathology

Samples were harvested and fixed in 4% paraformaldehyde then dehydrated and embedded in paraffin blocks to make tissue slices, and stain with H&E. Then the tissue slices of different groups of mouse organs in the same proportion were observed and photographed.

### Immunofluorescence Analysis

Immunofluorescence staining was employed to observe the expression of TNF‐α (HuaBio, Hangzhou), Occludin (HuaBio, Hangzhou), Claudin1 (HuaBio, Hangzhou), Nrf2 (HuaBio, Hangzhou), and iNOS (HuaBio, Hangzhou), CD86 (Cell Signaling Technology), CD206 (Cell Signaling Technology), F4/80 (Cell Signaling Technology). The colon sections were blocked with 5% BSA first, followed by incubation with primary antibodies and secondary antibodies as well as DAPI solution. After that, slides were dehydrated and imaged using Nikon microscope (Nikon ECLPSE 80i, Japan).

### Inflammatory Cell Infiltration

EGFP‐positive inflammatory cells were recovered from peritoneal fluid of EGFP mice in thioglycollate elicited peritonitis model.^[^ ^36^ ^]^ Healthy and IBD mice were pretreated with indicated DNA materials by rectal administration, and then transferred with EGFP‐positive inflammatory cells (10^5^ cells per mouse) by tail vein injection. After 24 h of injection. The colonic sections were counterstained with DAPI for fluorescence microscope observation.

### ​Flow Cytometry Analysis of Immune Cells from Colonic Mucosa​

Fresh colon tissue was flushed with CMF buffer, cut into 1 mm^3^ fragments, and incubated in CMF/FBS/DTE at 37 °C for 20 minutes. Cell suspension was collected and total immune cells were isolated in Percoll solution. Then total immune cells were stained with PE‐conjugated anti mouse TCRβ (Invitrogen), FITC‐conjugated anti mouse TCRγδ, and FITC‐conjugated anti mouse Gr‐1 (BD Biosciences), and analyzed by FACS.

### RNA‐Sequencing

Total RNA was extracted from mice colon tissue by TRIzol (Invitrogen, Thermo Fisher Scientific). Then libraries were constructed by Majorbio (Shanghai, China). After constructing the transcriptome library, sequencing was performed by the Illumina HiSeq xten/NovaSeq 6000 sequencer in Majorbio (Shanghai, China). Data analysis was performed using Majorbio biological cloud platform (https://cloud.majorbio.com/page/tools/).

### Statistical Analysis

All experiments were independently replicated at least three times. The mean ± standard deviation (SD) was used to report the results. The student's t‐test, one‐way analysis of variance (ANOVA) followed by Tukey post hoc analysis, or two‐way ANOVA test were used to determine the significant differences between the different groups. All data were analyzed by GraphPad Prism 9.0. Statistically significant *P* values were indicated in figures and legends as *P < 0.05, **P < 0.01, ***P < 0.001.

## Conflict of Interest

The authors declare no conflict of interest.

## Supporting information



Supporting Information

## Data Availability

The data that support the findings of this study are available in the supplementary material of this article.

## References

[1] J. Han , J. Ye , J. Shi , Y. Fan , X. Yuan , R. Li , G. Niu , M. Abubakar , Y. Kang , X. Ji , Adv. Funct. Mater. 2025, 35, 2413261.

[2] K. Wang , Q. Chen , L. Ding , Y. Zhu , X. Wang , M. Zhou , M. Chang , M. Pei , Y. Zhang , Y. Zhang , Y. Chen , H. Qin , Nano Today 2023, 50, 101876.

[3] Q. Guan , J. Immunol. Res. 2019, 16, 7247238. 10.1155/2019/7247238 PMC6914932 31886308

[4] K. Wei , F. Gong , J. Wu , W. Tang , F. Liao , Z. Han , Z. Pei , H. Lei , L. Wang , M. Shao , Z. Liu , L. C. Cheng , ACS Nano 2023, 17, 21539. 37843009 10.1021/acsnano.3c06551

[5] Q. Huang , Y. Yang , Y. Zhu , Q. Chen , T. Zhao , Z. Xiao , M. Wang , X. Song , Y. Jiang , Y. Yang , J. Zhang , Y. Xiao , Y. Nan , W. Wu , K. Ai , Small 2023, 19, 2207350. 10.1002/smll.202207350 36760016

[6] L. Li , P. Peng , N. Ding , W. Jia , C. Huang , Y. Tang , Antioxidants 2023, 12, 967. 37107341 10.3390/antiox12040967 PMC10135842

[7] Z. Cai , S. Wang , J. Li , Front. Med. 2021, 8, 765474. 10.3389/fmed.2021.765474 PMC8720971 34988090

[8] X. Cao , S. Tao , W. Wang , S. Wu , Y. Hong , X. Wang , Y. Ma , H. Qian , Z. Zha , Nat. Commun. 2024, 15, 8428. 39341804 10.1038/s41467-024-52722-7 PMC11438902

[9] D. Li , J. Li , T. Chen , X. Qin , L. Pan , X. Lin , W. Liang , Q. Wang , ACS Appl. Mater. Interfaces 2023, 15, 38273. 37530040 10.1021/acsami.3c06693

[10] Y. Gao , J. Zou , B. Chen , Y. Cao , D. Hu , Y. Zhang , X. Zhao , J. Wen , K. Liu , K. Wang , Biomater. Sci. 2023, 11, 618. 36484291 10.1039/d2bm01256a

[11] S. Zhao , Y. Li , Q. Liu , S. Li , Y. Cheng , C. Cheng , Z. Sung , Y. Du , C. Butch , H. Wei , Adv. Funct. Mater. 2020, 30, 2004692.

[12] H. Nakase , N. Sato , N. Mizuno , Y. Ikawa , Autoimmun. Rev. 2022, 21, 103017. 34902606 10.1016/j.autrev.2021.103017

[13] S. Zhang , J. Ermann , M. Succi , A. Zhou , M. Hamilton , B. Cao , J. Korzenik , J. Glickman , P. Vemula , L. Climcher , G. Traverso , R. Langer , J. Karp , Sci. Transl. Med. 2025, 7, 300ra128. 10.1126/scitranslmed.aaa5657 PMC4825054 26268315

[14] L. Hong , G. Chen , Z. Cai , H. Liu , C. Zhang , F. Wang , Z. Xiao , J. Zhong , L. Wang , Z. Wang , W. Cui , Adv. Sci. 2022, 9, 2200281. 10.1002/advs.202200281 PMC9284187 35524641

[15] E. Kunkel , B. J. , C. Eugene , Immunity 2002, 16, 1. 11825560 10.1016/s1074-7613(01)00261-8

[16] X. Ren , L. D. Manzanares , E. B. Piccolo , J. M. Urbanczyk , D. P. Sullivan , L. K. Yalom , T. M. Bui , C. Lantz , H. Najem , P. S. Dulai , A. B. Heimberger , E. B. Thorp , R. Sumagin , J. Clin. Invest. 2023, 133, 170733. 10.1172/JCI170733 PMC10378177 37261911

[17] B. Dai , J. Hackney , R. Ichikawa , A. Nguyen , J. Elstrott , L. Orozco , K. Sun , Z. Modrusan , A. Gogineni , A. Scherl , J. Gubatan , A. Habtezion , M. Deswal , M. Somsouk , W. Faubion , A. Chai , Z. Sharafali , A. Hassanali , Y. Oh , S. Tole , J. McBride , M. Keir , T. Yi , Cell Rep. Med. 2021, 2, 100381. 34467254 10.1016/j.xcrm.2021.100381 PMC8385326

[18] Y. Song , M. Yuan , Y. Xu , H. Xu , Pharmaceuticals 2022, 15, 1080. 36145301 10.3390/ph15091080 PMC9502105

[19] Y. Zhang , Z. Wu , Q. Zhao , Y. Liu , Q. Haung , M. Zhang , S. Li , D. Wang , N. Li , Y. Chi , Y. Liu , Biology 2024, 13, 322. 38785804 10.3390/biology13050322 PMC11117591

[20] W. Wang , R. Yan , L. Lin , L. Peng , Y. Xiong , H. Chen , X. Gao , K. Liu , L. Zhou , Y. Lin , Chem. Eng. J. 2024, 493, 1527736.

[21] H. Wang , J. Gong , W. Chen , Q. Sun , T. Zhang , Y. Lin , X. Cai , Nano Today 2024, 56, 102252.

[22] Y. Xie , S. Shi , W. Lv , X. Wang , L. Yue , C. Deng , D. Wang , J. Han , T. Ye , Y. Lin , ACS Nano 2024, 18, 26704. 39276332 10.1021/acsnano.4c06598

[23] M. Zhou , S. Gao , X. Zhang , T. Zhang , T. Zhang , T. Tian , S. Li , Y. Lin , C. X. Zhang , Bioact. Mater. 2020, 6, 1676. 33313447 10.1016/j.bioactmat.2020.11.018 PMC7708773

[24] D. Yan , C. Huang , W. Ouyang , J. Hu , Z. Liu , Adv. Healthcare Mater. 2024, 13, 2400198. 10.1002/adhm.202400198 39073031

[25] Y. Zhao , S. Li , M. Feng , M. Zhang , Z. Liu , Y. Yao , T. Zhang , Y. Jang , Y. Lin , X. Cai , Small 2023, 19, 2302326. 10.1002/smll.202302326 37317020

[26] Y. Xu , Z. Yan , Y. Ma , H. Ding , Int. J. Biol. Macromol. 2024, 257, 128703. 38072351 10.1016/j.ijbiomac.2023.128703

[27] Y. Wei , K. Wang , S. Luo , F. Li , X. Zuo , C. Fan , C. Fan , Q. Li , Small 2022, 18, 2107640. 10.1002/smll.202107640 35119201

[28] X. Yan , B. Yang , Y. Chen , Y. Song , J. Ye , Y. Pan , B. Zhou , Y. Wang , F. Mao , Y. Dong , D. Liu , J. Yu , Adv. Mater. 2021, 33, 2104758. 10.1002/adma.202104758 34657320

[29] S. Moench , P. Lemke , A. Hansen , C. Bickmann , M. Peng , K. Rabe , C. Domínguez , C. Niemeyer , Angew. Chem., Int. Ed. 2025, 64, 202414480. 10.1002/anie.202414480 39420772

[30] C. Yao , R. Zhang , J. Tang , D. Yang , Nat. Protoc. 2021, 16, 5460. 34716450 10.1038/s41596-021-00621-2

[31] M. Zhang , X. Zhang , T. Tian , Q. Zhang , Y. Wen , J. Zhu , D. Xiao , W. Cui , Y. Lin , Bioact. Mater. 2021, 8, 368. 34541407 10.1016/j.bioactmat.2021.06.003 PMC8429917

[32] X. Chen , J. He , Y. Xie , T. Zhang , S. Li , Y. Zhao , N. Hu , X. Cai , Cell. Prolif. 2023, 56, 13424. 10.1111/cpr.13424 PMC10392044 36802079

[33] P. Liu , Y. Zhao , X. Liu , J. Sun , D. Xu , Y. Li , Q. Li , L. Wang , S. Yang , C. Fan , J. Lin , Angew. Chem. Int. Ed. Engl. 2018, 57, 5418. 29528530 10.1002/anie.201801498 PMC6142180

[34] T. Sutherland , D. Dyer , J. Allen , Science 2023, 379, abp8964. 10.1126/science.abp8964 36795835

[35] S. Yang , Y. Cheng , M. Liu , J. Tang , S. Li , Y. Huang , X. Kou , C. Yao , D. Yang , Nano Today 2024, 56, 102224.

[36] X. Zhang , L. Song , L. Li , B. Zhu , L. Huo , Z. Hu , X. Wang , J. Wang , M. Gao , J. Zhang , Z. Hua , Signal Transduct Target Ther. 2021, 6, 235. 34131110 10.1038/s41392-021-00626-z PMC8206212

