# Supplementary material for: A Charge‐Adhesive Targeted DNA Gel Bandage for the Precision Treatment of Inflammatory Bowel Disease
Source: Adv Sci (Weinh). 2025 Jul 20;12(38):e09419. doi: 10.1002/advs.202509419 (PMC12520488; doi:10.1002/advs.202509419)

Supporting Information

Title A Charge-Adhesive Targeted DNA Gel Bandage for the Precision Treatment of Inflammatory Bowel Disease

*Peifen Lu, Hongxiu Yuan, Gang Wang, Tao Cheng, Lie Li, Yixi Dong, Runyu Zhao, Xuerui Zhang^*^, Jianwei Jiao^*^, and Jin Jiao^*^*

**Table of Contents**

**Table S1** Oligonucleotide sequences used in this work…………………...........…..…...….....3

**Table S2** Primer sequences for RT-qPCR analysis…………………………..…..........….….....4

**Figure S1** Biocompatibility of DNAgb……………………..……………..…………...............5

**Figure S2** mRNAs of pro-inflammation cytokines IL-6 and IL-1β………..…...........………....6

**Figure S3** The cellular uptake efficiency of TDN-ApITGA4 and DNAgb….…...........……..…7

**Figure S4** The stability of TDN-ApITGA4 and DNAgb……………………....…...........…..…8

**Figure S5** Flow cytometry showed the targeting ability of ITGA4 aptamers…..……............…9

**Figure S6** Zeta potentials of DNAgb and DNAgbmut with or without MgCl_2_...…..........….....10

**Figure S7** Fluorescence images of of IBD mice after rectal administration of the DNAgb^mut^ and DNAgb with or without MgCl_2_. ……………………………….............................…........…...11

**Figure S8** The degree of oxidative stress in colon tissues………………...………..........…..12

**Figure S9** In vivo biocompatibility………………………………………………...........…...13

**Figure S10** mRNA level of TNF-α, IL-6, and IL-1β of colon tissues on IBD mice under different treatment………………………………………………………...……….............….14

**Figure S11** Immunofluorescence staining of M2 macrophage subtypes (F4/80^+^ CD206^+^) in colon tissues from IBD mice subjected to different treatments………….…..............…….…15

**Figure S12** Flow cytometric analysis of T cells of colonic mucosa on IBD mice under DNAgb treatment………………………………………………………..............….............……….…16

**Original data:**

**Original data for blot**………………………………………………..…...….............…….…17

**Original data for microscope images**……………………………….…...…................….…18

**Gate information for flow cytometry**………………………………....….............……....…20

**Table S1. Oligonucleotide sequences used in this work.**

| **Name** | **Sequence (5’-3’)** |
| --- | --- |
| Primer-1 | TAGGAACATCAAACGACAGCCA |
| Template-1 | /Phosphate/TCGTTTGATGTTCCTATTTTTTTTTTTTTTTTTTTTCGGGAAGAGCATGCCCATCCTCGTATCACCAGGCAGTTGAGTCATGCGAGGGTCCAATACCGTTTTTTTTTTTTTTTTTTTTTTTGGCTG |
| Primer-2 | TGGCTGTCGTTTGATGTTCCTA |
| Template-2 | /Phosphate/CAGCCATTTTTTTTTTTTTTTTTTTTCGGGAAGAGCATGCCCATCCTCGTATCACCAGGCAGTTGAGTCATGCGAGGGTCCAATACCGTTTTTTTTTTTTTTTTTTTTTTTAGGAACATCAAACGA |
| S1 | ATTTATCACCCGCCATAGTAGACGTATCACCAGGCAGTTGAGACGAACATTCCTAAGTCTGAA |
| S2 | ACATGCGAGGGTCCAATACCGACGATTACAGCTTGCTACACGATTCAGACTTAGGAATGTTCG |
| S3-ApITGA4 | AGATGGGGTTTAGGTGAATTGAAGTGGCTTCATTTAGTAGGGTGCTAGCATTTTTTTTACTACTATGGCGGGTGATAAAACGTGTAGCAAGCTGTAATCGACGGGAAGAGCATGCCCATCC |
| S4 | ACGGTATTGGACCCTCGCATGACTCAACTGCCTGGTGATACGAGGATGGGCATGCTCTTCCCG |
| FAM-S4 | FAM-ACGGTATTGGACCCTCGCATGACTCAACTGCCTGGTGATAC  GAGGATGGGCATGCTCTTCCCG |
| ApITGA4 | AGATGGGGTTTAGGTGAATTGAAGTGGCTTCATTTAGTAGGGTGCTAGCAT |
| FAM-ApITGA4 | FAM-AGATGGGGTTTAGGTGAATTGAAGTGGCTTCATTTAGTAGG  GTGCTAGCAT |

Note: The underlined sequences with red represent the Integrin α4 aptamer (ApITGA4).

**Table S2. Primer sequences for RT-qPCR analysis.**

| **Gene** | **Sequence (5’-3’)** |
| --- | --- |
| ACTB (F) | GTGACGTTGACATCCGTAAAGA |
| ACTB (R) | GCCGGACTCATCGTACTCC |
| TNF-α (F) | CAGGCGGTGCCTATGTCTC |
| TNF-α (R) | CGATCACCCCGAAGTTCAGTAG |
| iNOS (F) | GTTCTCAGCCCAACAATACAAGA |
| iNOS (R) | GTGGACGGGTCGATGTCAC |
| HO-1 (F) | AGGTACACATCCAAGCCGAGA |
| HO-1 (R) | CATCACCAGCTTAAAGCCTTCT |
| IL-6 (F) | CTGCAAGAGACTTCCATCCAG |
| IL-6 (R) | AGTGGTATAGACAGGTCTGTTGG |
| IL-1β (F) | GAAATGCCACCTTTTGACAGTG |
| IL-1β (R) | TGGATGCTCTCATCAGGACAG |


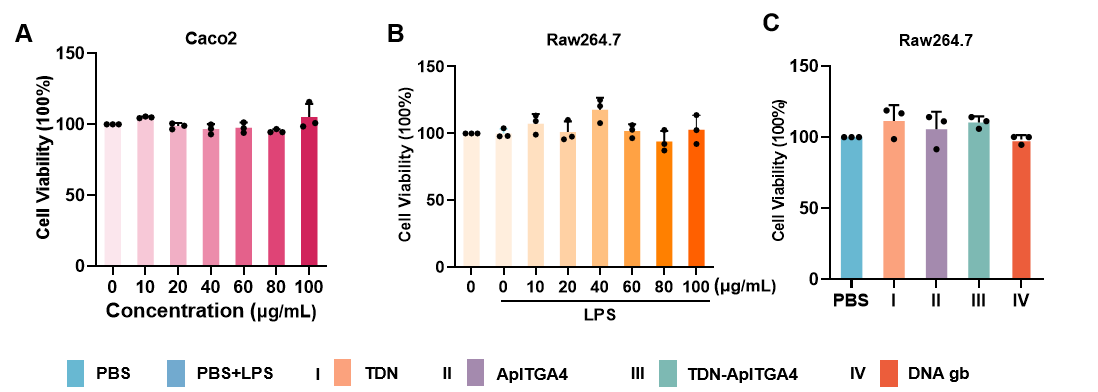


**Figure S1:** Biocompatibility of DNAgb. (A) Cell viability of Caco2 cells treated with different concentrations of DNAgb. (B) Effect of different concentrations of DNAgb on LPS-stimulated Raw264.7 cells. (C) Cell viability of Raw264.7 cells after various treatment. The data are presented as the means ± SD, n = 3.


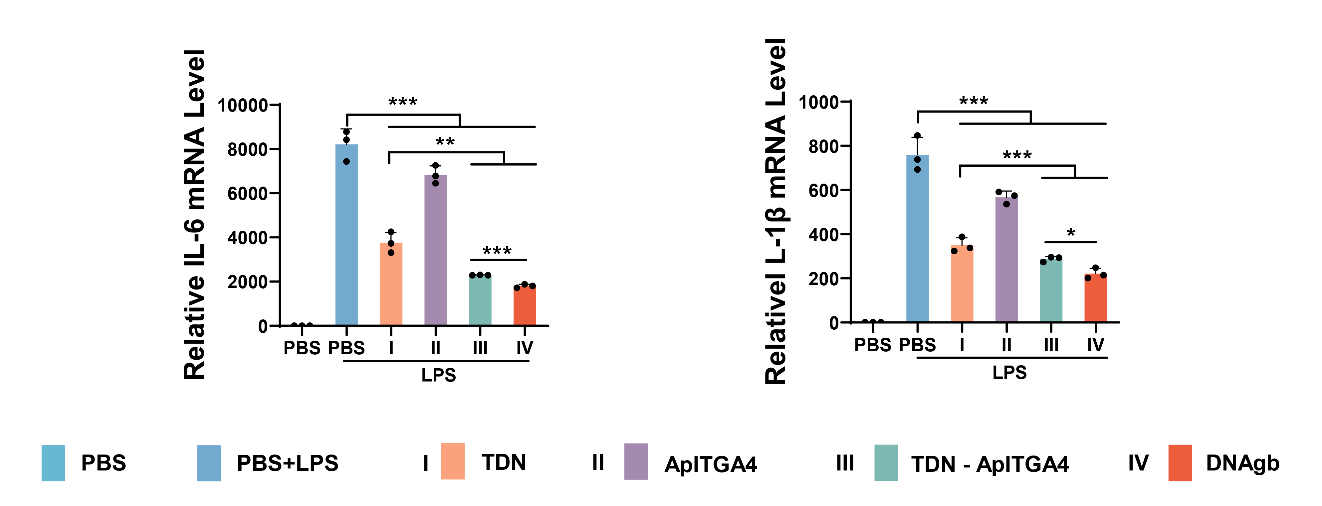


**Figure S2:** mRNAs of pro-inflammation cytokines IL-6 and IL-1β. mRNAs of pro-inflammation cytokines IL-6 (A) and IL-1β (B) were quantified by real-time PCR. Data are presented as mean ± SD, n = 3. *P < 0.05, **P < 0.01, ***P < 0.001.


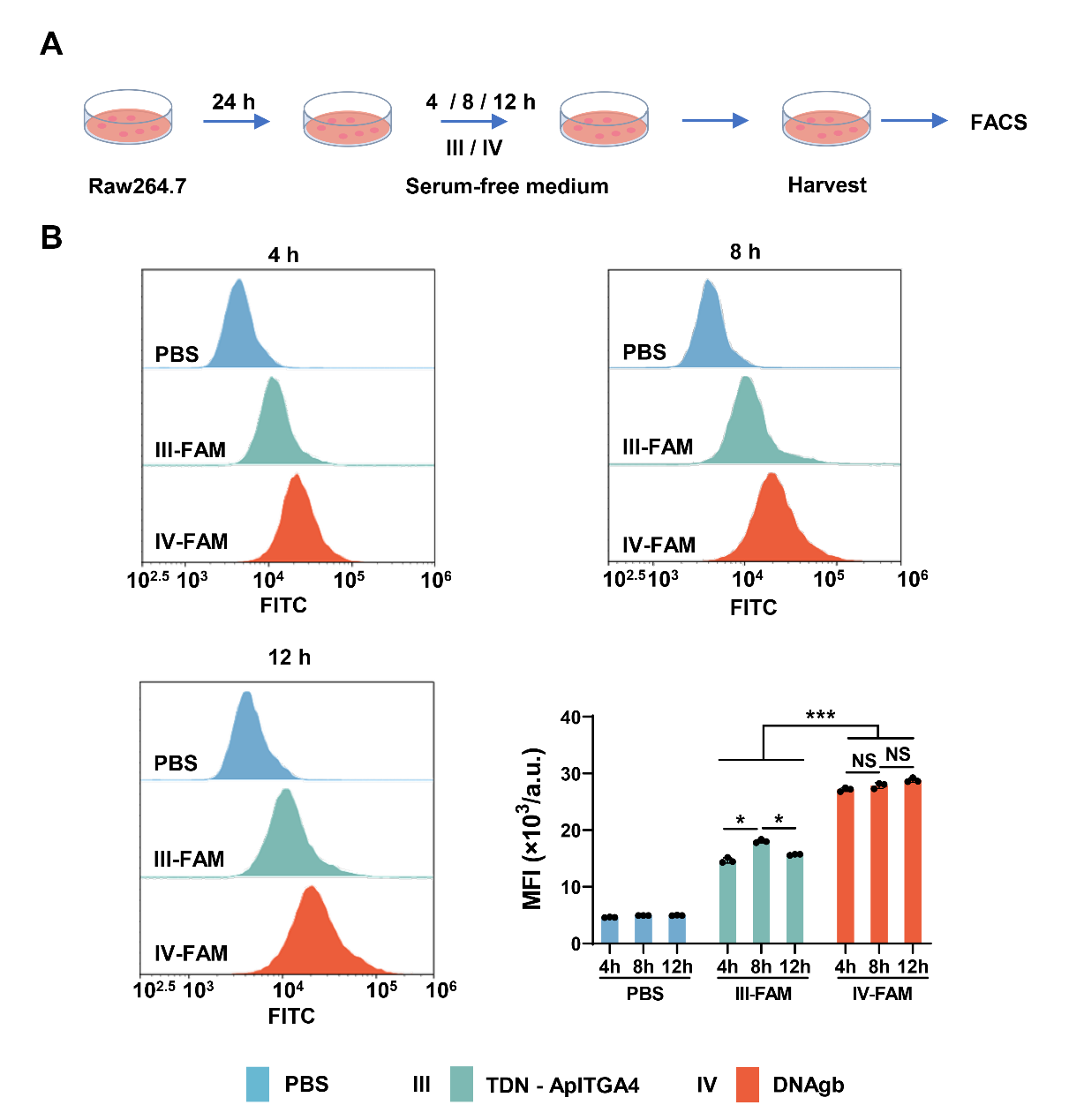


**Figure S3:** The cellular uptake efficiency of TDN-ApITGA4 and DNAgb. (A) The illustration of cell experiment. (B) The cellular uptake efficiency of TDN-ApITGA4 and DNAgb in Raw264.7 with different time. Data are presented as mean ± SD, n = 3. *P < 0.05, ***P < 0.001, NS, not significantly.


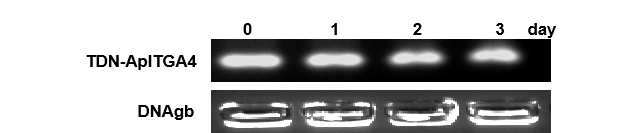


**Figure S4:** The stability of TDN-ApITGA4 and DNAgb in simulated intestinal fluid (SIF) for different times.


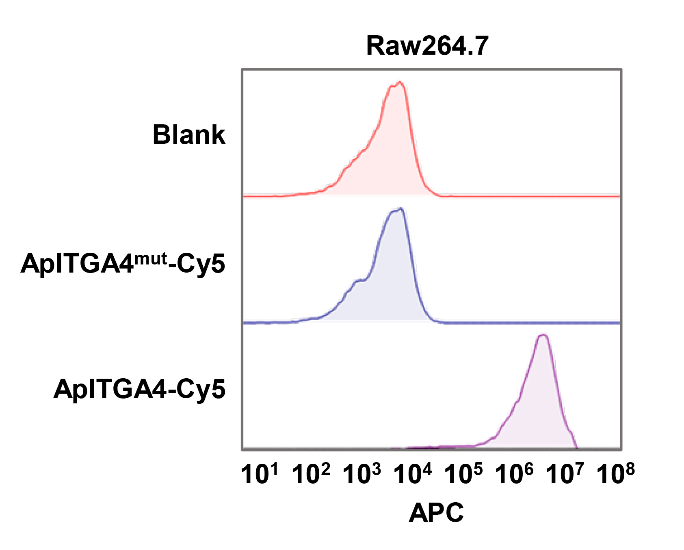


**Figure S5** Flow cytometry showed the targeting ability of ITGA4 aptamers. n = 3.


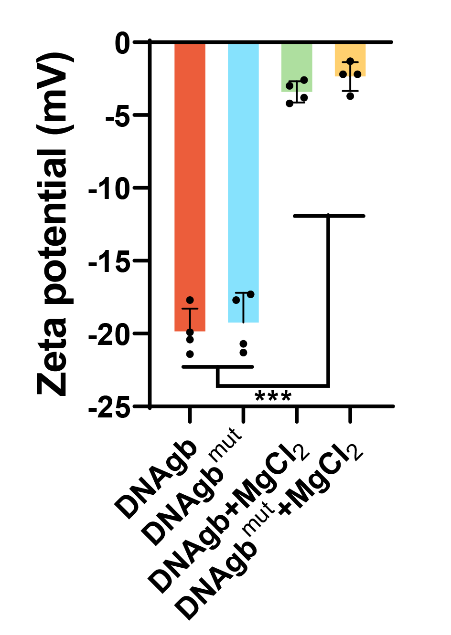


**Figure S6:** Zeta potentials of DNAgb and DNAgb^mut^ with or without MgCl_2_. Data are presented as mean ± SD, n = 4.


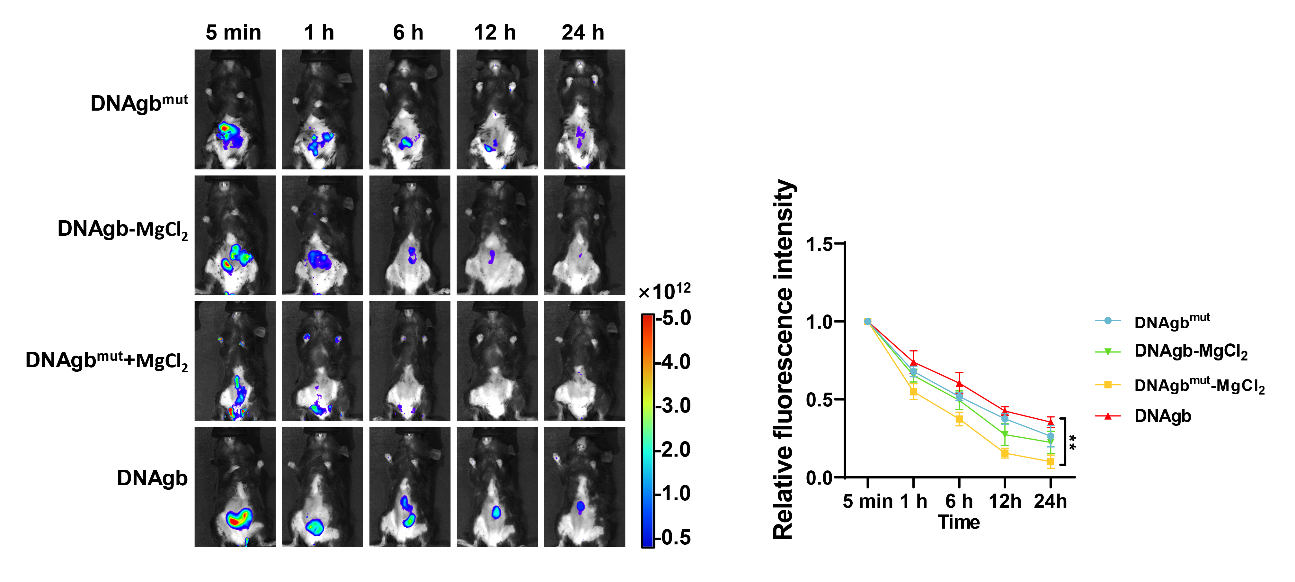


**Figure S7:** Fluorescence images of IBD mice after rectal administration of the DNAgb^mut^ and DNAgb with or without MgCl_2_, and it’s quantified for Cy5 fluorescence signal. The data are presented as the mean ± SD, n = 3. **P < 0.01.


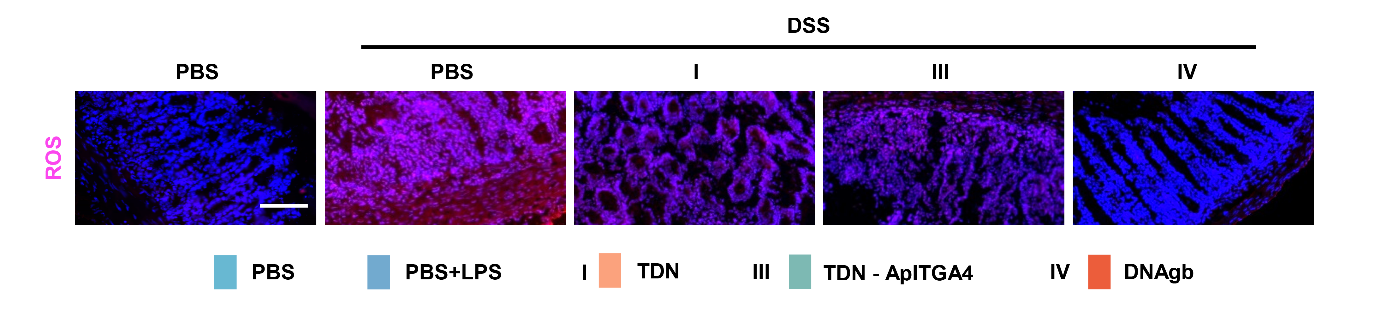


**Figure S8:** The degree of oxidative stress in the colon tissues of each group with representative dihydroethidium (DHE) staining (red fluorescence). Scale bar: 100 μm.


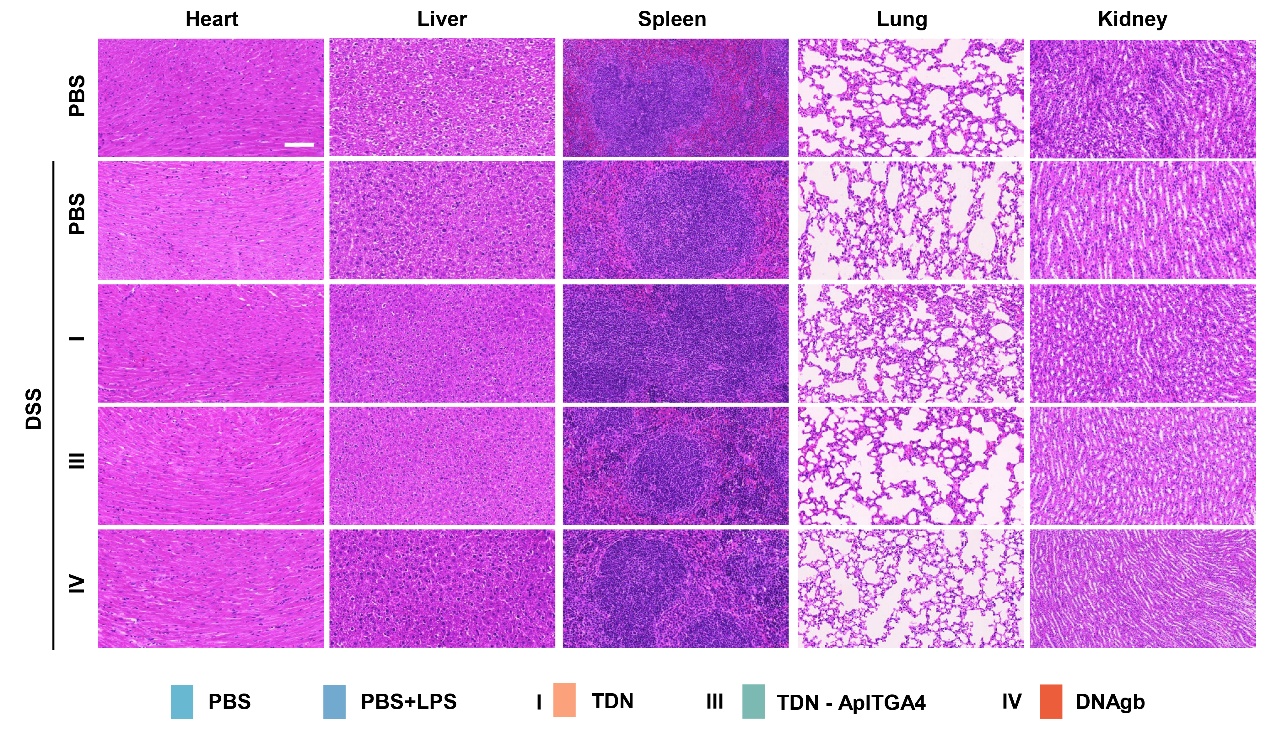


**Figure S9:** In vivo biocompatibility. H&E staining of tissues from the heart, lung, liver, and kidney of each group showed the biocompatibility of TDN, TDN-ApITGA4, DNAgb in mice. Scale bar: 100 µm.


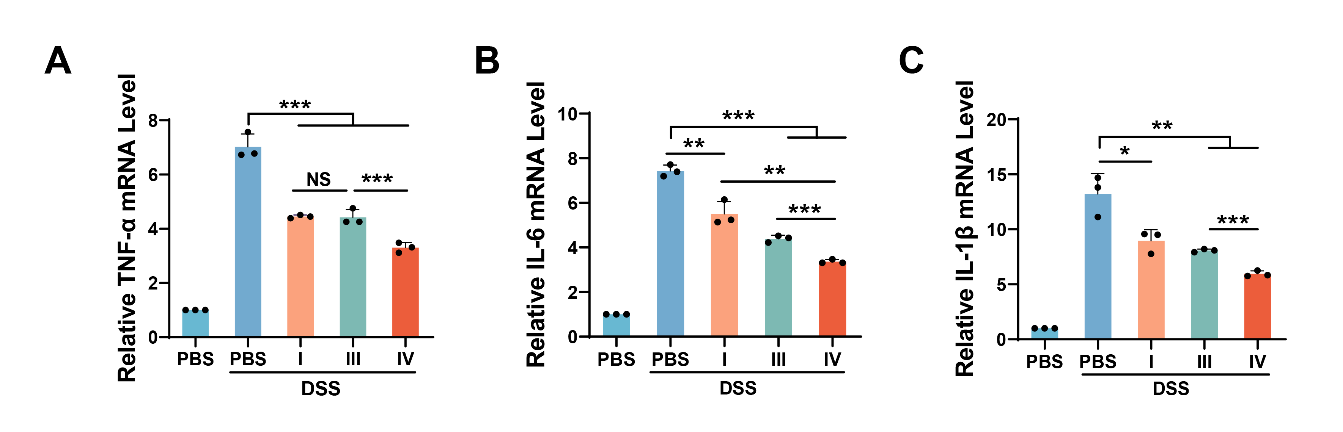


**Figure S10** mRNA level of TNF-α (A), IL-6 (B), and IL-1β (C) of colon tissues on IBD mice under different treatment. Data are presented as mean ± SD, n = 3. *P < 0.05, **P < 0.01, ***P < 0.001.


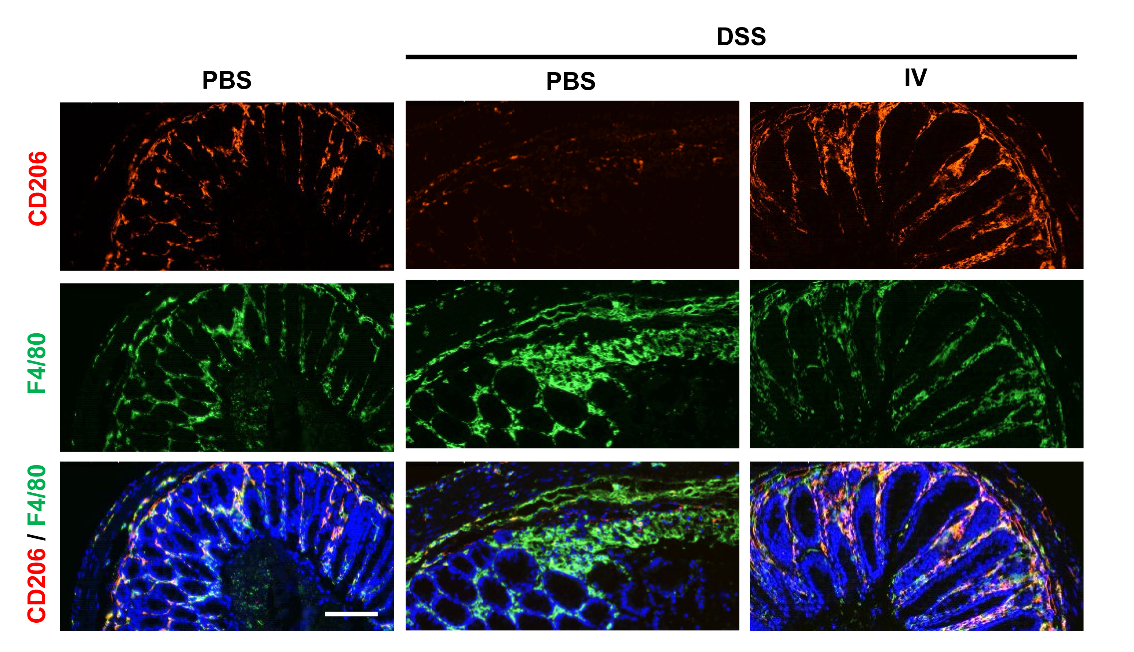


**Figure S11** Immunofluorescence staining of M2 macrophage subtypes (F4/80^+^ CD206^+^) in colon tissues from IBD mice subjected to different treatments. n = 3.


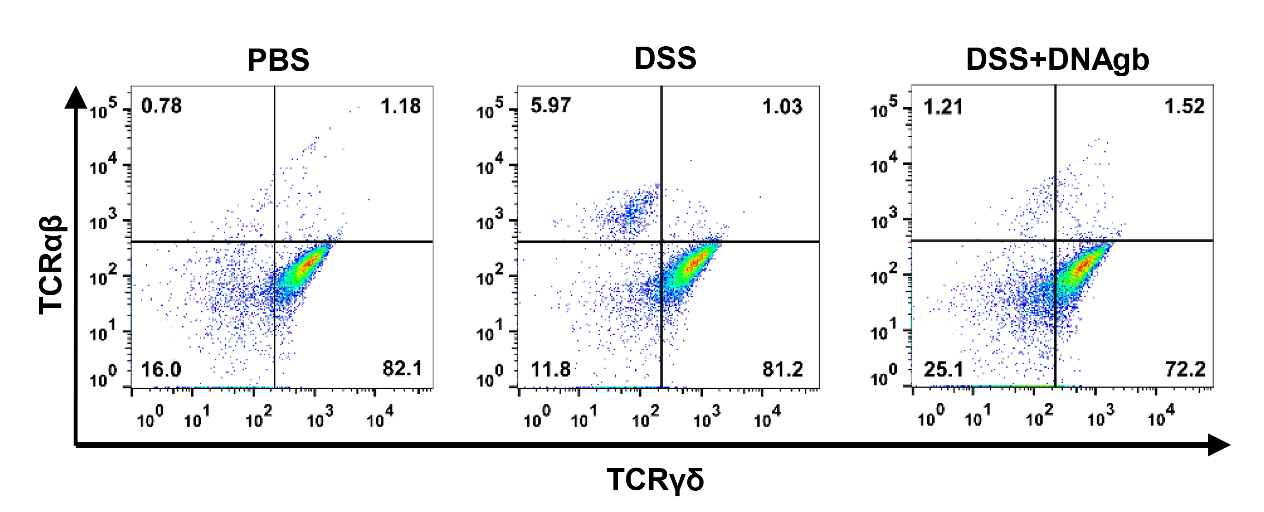


**Figure S12** Flow cytometric analysis of T cells of colonic mucosa on IBD mice under DNAgb treatment. n = 3.

**Original data for blot**


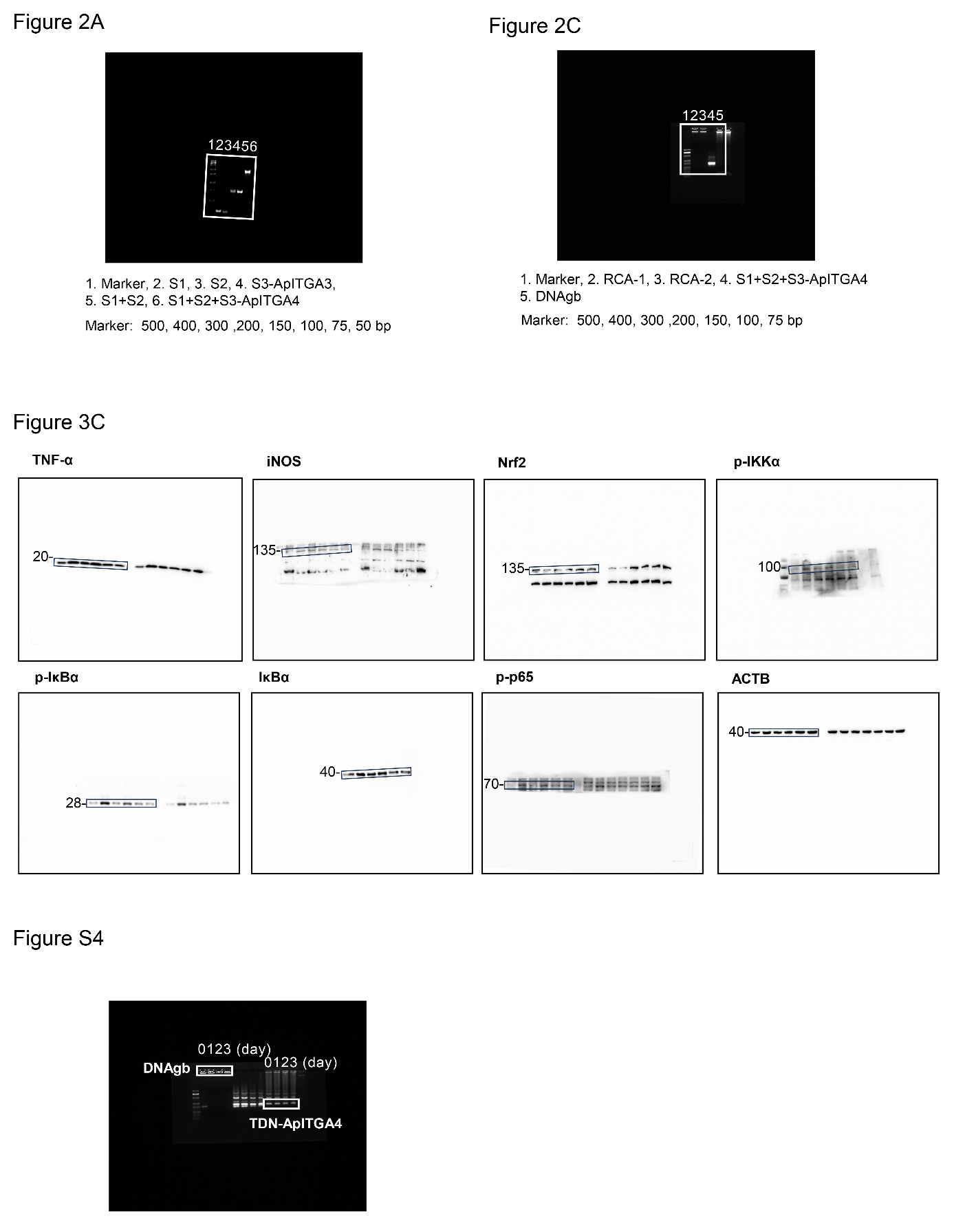


**Original data for microscope images**


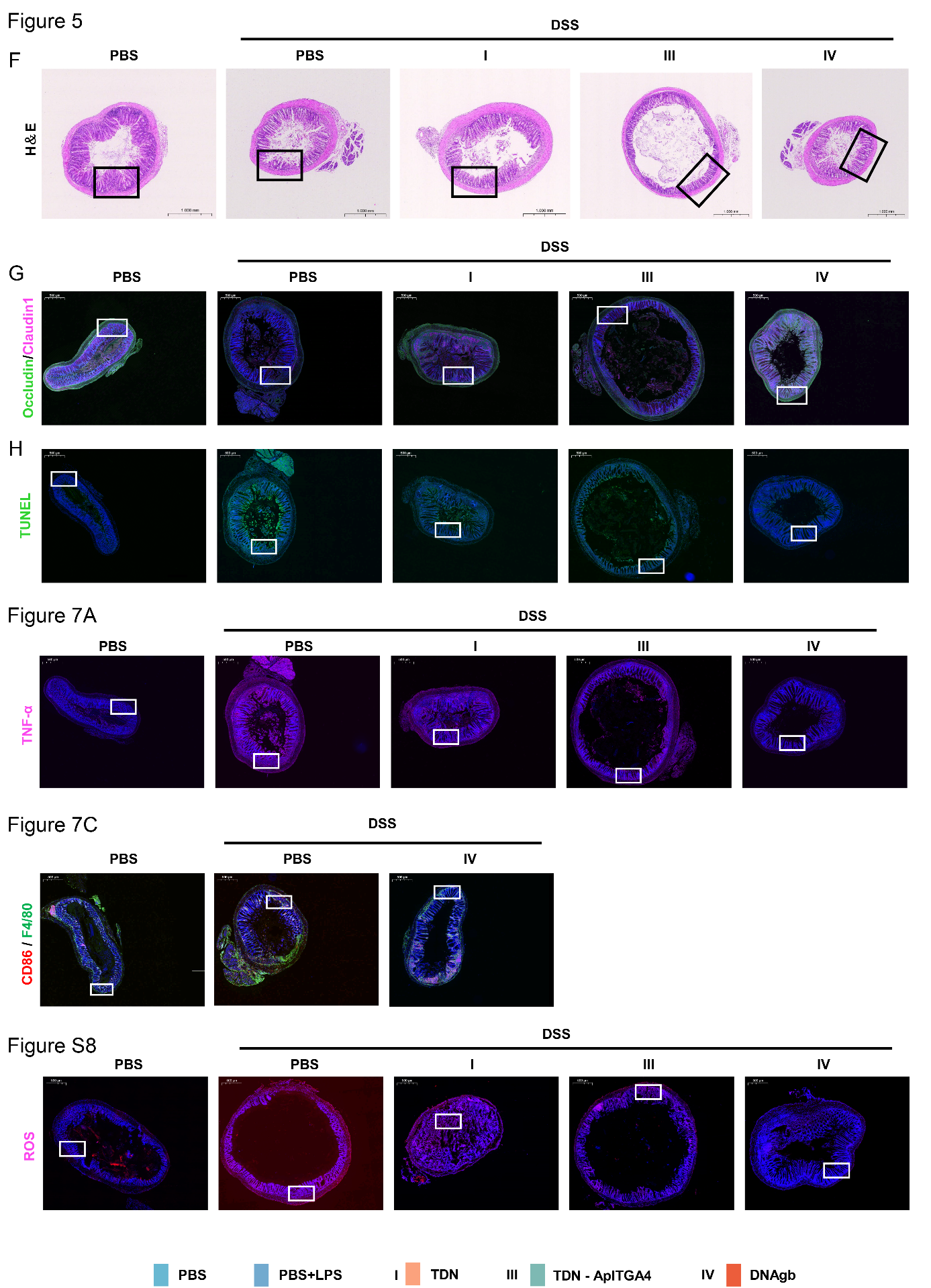


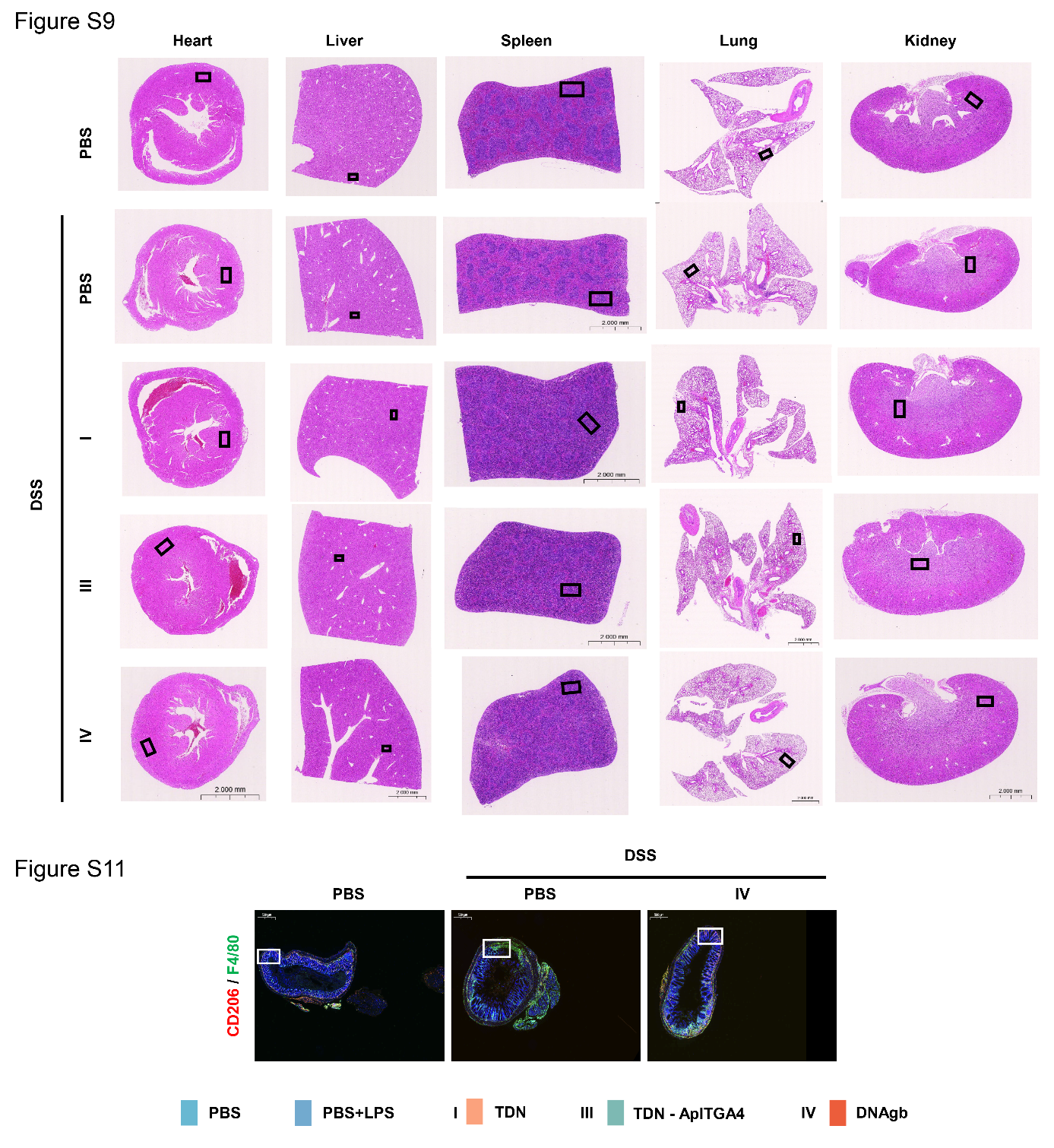


**Gate information for flow cytometry**


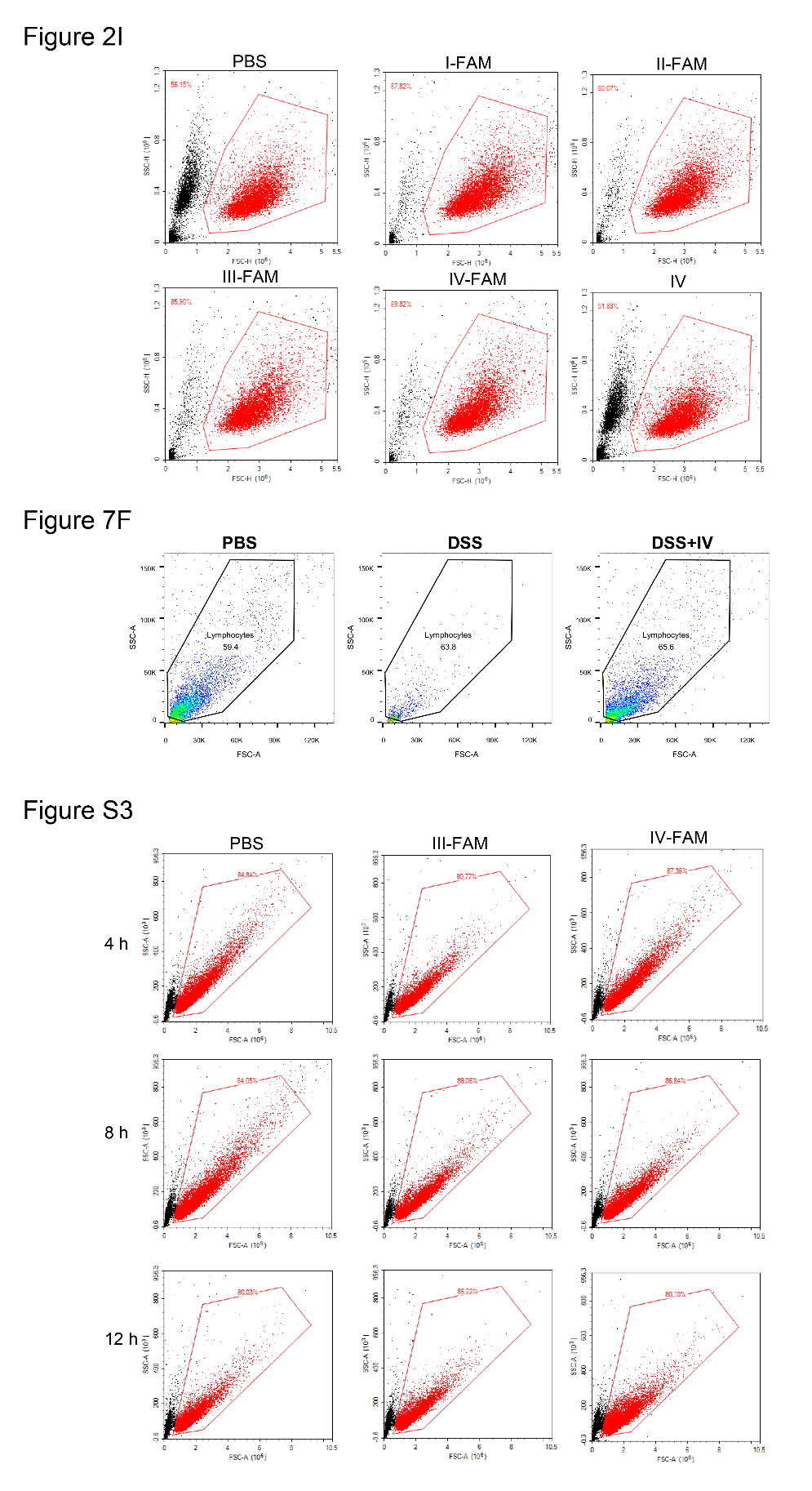


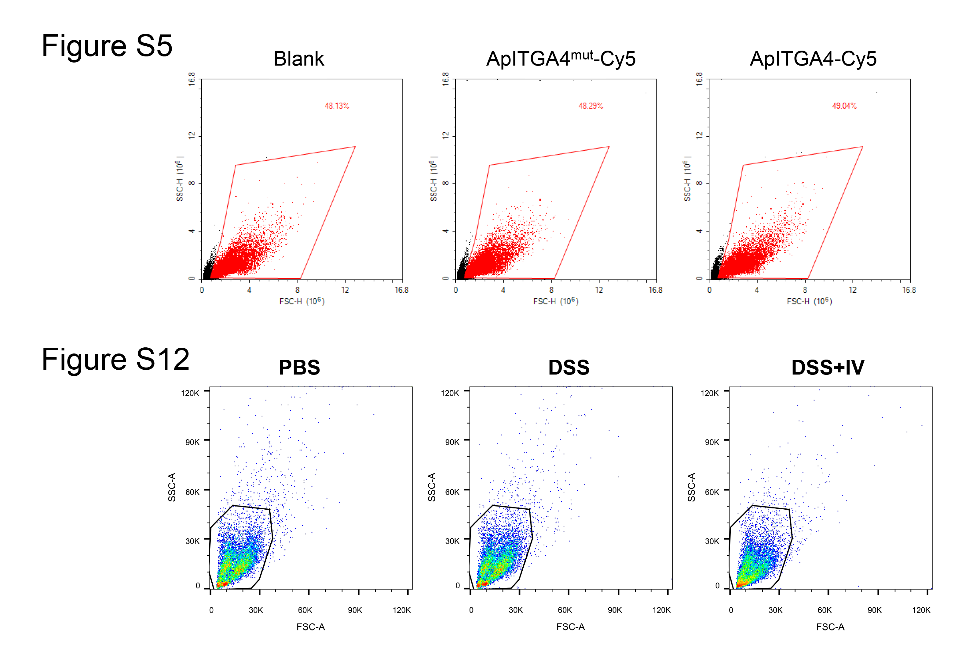

Supplement: Supplementary file 1 — Supporting Information [file ADVS-12-e09419-s001.docx]
